# Data-Driven AI Models within a User-Defined Optimization Objective Function in Cement Production

**DOI:** 10.3390/s24041225

**Published:** 2024-02-14

**Authors:** Othonas Manis, Michalis Skoumperdis, Christos Kioroglou, Dimitrios Tzilopoulos, Miltos Ouzounis, Michalis Loufakis, Nikolaos Tsalikidis, Nikolaos Kolokas, Panagiotis Georgakis, Ilias Panagoulias, Alexandros Tsolkas, Dimosthenis Ioannidis, Dimitrios Tzovaras, Mile Stankovski

**Affiliations:** 1 Titan Cement Group, 11143 Athens, Greece; o.manis@titancement.com (O.M.); p.georgakis@titancement.com (P.G.); i.panagoulias@titancement.com (I.P.); a.tsolkas@titancement.com (A.T.); 2 Centre of Research & Technology—Hellas (CERTH), Information Technologies Institute, 57001 Thessaloniki, Greece; ckioro@iti.gr (C.K.); dimtzilop@iti.gr (D.T.); mloufakis@iti.gr (M.L.); ntsalikidis@iti.gr (N.T.); nikolokas@iti.gr (N.K.);; 3 Faculty of Electrical Engineering and Information Technologies, Cyril and Methodius University, 1000 Skopje, North Macedonia; milestk@feit.ukim.edu.mk

**Keywords:** optimization, feature selection, machine learning, clustering, differential evolution, cement mill, cement kiln, key performance indicator

## Abstract

This paper explores the energy-intensive cement industry, focusing on a plant in Greece and its mill and kiln unit. The data utilized include manipulated, non-manipulated, and uncontrolled variables. The non-manipulated variables are computed based on the machine learning (ML) models and selected by the minimum value of the normalized root mean square error (*NRMSE*) across nine (9) methods. In case the distribution of the data displayed in the user interface changes, the user should trigger the retrain of the AI models to ensure their accuracy and robustness. To form the objective function, the expert user should define the desired weight for each manipulated or non-manipulated variable through the user interface (UI), along with its corresponding constraints or target value. The user selects the variables involved in the objective function based on the optimization strategy, and the evaluation is based on the comparison of the optimized and the active value of the objective function. The differential evolution (DE) method optimizes the objective function that is formed by the linear combination of the selected variables. The results indicate that using DE improves the operation of both the cement mill and kiln, yielding a lower objective function value compared to the current values.

## 1. Introduction

The present-day trend of energy preservation and emissions reduction has had an impact on the cement sector as well. The incorporation of cement has emerged as an essential element in modern construction practices, as it contributes significantly to enhancing the overall quality of building projects. The determination of cement type and quality is reliant on the measurement of cement fineness, which is determined by the Blaine index and the residue index. The level of fineness in cement directly influences the speed of hydration and the development of strength. As the particle size decreases, there is an increase in the surface area-to-volume ratio, facilitating a greater interaction between water and cement. As a consequence, there is an accelerated response with water, leading to a rapid increase in both strength development and heat generation during the hydration process. Extensive research has been conducted to optimize separate processes in both the mill and kiln. This has been achieved through the application of optimization techniques. In work [1], an energy balance analysis is produced on the basis of raw materials and product process employing an analytic hierarchy process (AHP) global optimizer and a multi-criteria decision method. The optimization of both energy and clinker compounds reduce energy consumption. The research [2] focuses on mill load analysis considering mechanical vibration and acoustic signals, employing intelligent selective ensemble modeling. The work [3] aimed to determine the best production method, estimate profits, and analyze processing time and raw material usage. The goal programming (GP) model is utilized to optimize the four objectives. Study [4] aims to optimize a ten (10)-objective problem, including economic cost, energy, C$O_{2}$, particulate matter (PM), nitric oxide (NOx) emissions, and heavy metals. Balancing these objectives is the main challenge, and the optimal values result from combining Spearman’s correlation, technique for order of preference by similarity (TOPSIS), conservation supply curves, and quadrant methods. In work [5], a real cement plant embraces the proposed predictive control system, and consequently, the system performance attains both economic benefits and the minimization of environmental noxious emissions. According to a study conducted [6], it was determined that increasing the grinding circuit capacity within the mill by 12–20% results in a decrease in overall specific energy consumption. The work [7] aims to find a temporary optimal operating point that is recomputed at each iteration using a cement mill’s dynamic model to approximate linearization.

However, the current research efforts have also focused on the integration of AI models in the optimization process of cement production. The study [8] revealed a direct relationship between feed quantity and composition, cement residue increase, and a decrease in Blaine fineness. In [9], the hardness in a cement mill is estimated. The nonlinear cement mill is modeled by Takagi-Sugeno modeling considering unmeasurable premise variables. Using this model, a proposition is made for a nonlinear observer to estimate state variables and clinker hardness. The work [10] centers around the utilization of a Markov chain model to generate the optimal particle size distribution (PSD) in an industrial mill, taking into account Blaine and residue indices. The study [11] emphasizes that AI-driven fault analysis should evaluate each model based on factors such as data source, data acquisition, data fusion, algorithm selection, and optimization. The study [12] explored the utilization of the fuzzy inference system (FIS) and the adaptive neuro-fuzzy inference system (ANFIS) for modeling and simulation of optimal production quantity and machine fault prediction. This approach integrates the benefits of rule-based fuzzy systems with the learning capabilities offered by neural networks. In the study [13], five (5) ML models were employed to optimize the cubic compressive strength (CCS) of the mix ratio design of magnesium phosphate cementitious composites (MPCC) by 55% to 85% over a one-month period. These models included particle swarm optimization of back propagation artificial neural network (PSO-BP-ANN), random forest (RF), decision tree (DT), linear regression (LR), and support vector machine (SVM). The work [14] aims to put forth a framework for the optimization of concrete mixture proportions. To achieve this, multi-objective optimization (MOO) are utilized, alongside ML models. These ML models are developed using K-fold cross-validation, Bayesian hyperparameter optimization, regression feature elimination, and the constrained–targeted adaptive evolutionary algorithm (C-TAEA). The work [15] demonstrates the use of ML models in the design of concrete based on construction and demolition waste. Despite the advantages in terms of sustainability, the design and material proportions for this type of concrete rely on non-linear relationships discovered through ML models. In [16], the water–cement ratio is explored, revealing that reduced cement content concrete takes three months, rather than one month, to achieve full strength. The achievement of optimizing the design of the concrete mix of structural elements is made possible through the utilization of ML models, specifically the artificial neural network (ANN) and regression algorithms. The studies conducted in the cement kiln have primarily concentrated on two key areas: the utilization of industrial waste materials like kiln dust and the reduction of pollutant emissions, specifically targeting the reduction of NOx levels. The examination carried out in [17] focuses on determining the optimal content of cement kiln dust (CKD) in cement substitution. The determination of the optimal CKD content and estimation of compressive strength in CKD-modified cement mortar was based on three distinct models: multiple expression programming (MEP), nonlinear regression (NLR), and an ANN. In [18], the reduction of NOx emissions in the kiln is achieved by using adaptive neural inference systems and genetic algorithms (ANFIS-GA).

The previous research on the utilization of ML models, along with optimization methods in cement production, has primarily concentrated on optimizing material proportions in smaller sub-processes and chemical reactions, rather than predicting parameters in larger processes. In addition, the proposed methods often neglect to consider the diverse nature of the sensor data. The optimization of the primary components of the flowsheet in the cement plant is a crucial matter, as it will concurrently optimize the specific parameters of the sub-functions within the system. More specifically, the operation of the mill is challenging due to its complex mechanism, constant changes in slurry composition, and raw material grinding. In addition, overloading or underloading the mill can damage the equipment over time, depending on the operator and material strength [2]. The mill operator plays a critical role in optimizing the mill’s energy consumption, cement quality, and equipment lifespan. ML models with optimized parameters are used to express the non-linear relationships. However, this is not sufficient as optimized operation of the cement plant is not guaranteed. For this reason, it is important to introduce the ML models into an optimization function to derive a final optimized operation.

The primary intuition behind this work is the acknowledgment that the correlations between dependent and independent variables in cement production are non-linear and dynamic. Therefore, the integration of AI models, along with their continuous retraining when data behavior changes, in an optimization method, can develop a robust cement production optimization system. Moreover, it is crucial to take into account several factors such as environmental impact, production cost, energy consumption, and more. This highlights the importance for cement plants to regulate their production in accordance with the preferences of the plant management while also complying with legal regulations regarding environmental protection and energy saving.

In this work, the system operator determines the objective function to be optimized rather than having a predetermined form. Once we have available data on the logger and the kiln, we will focus on these two sub-modules. To be more precise, the operator establishes whether a parameter is to be within predetermined bounds or converge to a desired value. It is also defined whether the optimization will be maximized or minimized. The data are extracted from a Mongo database that contains information about the Greek cement factory’s kiln or mill. The objective is to predict seven (7) non-manipulated variables for the mill and three (3) non-manipulated variables for the kiln. The manipulated variables are those that the user can determine, whereas the non-manipulated variables are those that are calculated using linear or non-linear relationships between the manipulated variables. Since there are fifty-four (54) features for the mill and twenty (20) features for the kiln, two feature selection algorithms named recursive feature elimination with cross-validation (RFECV) and sequential feature selector (SFS) are used to select the most suitable features for training the ML models. More specifically, the best features are the common features selected by these two algorithms. ML models are used to compute the non-manipulated variables because it is well known that the relationships between parameters in a complex process are almost unlikely to be linear. The optimal model is selected from among the regressors KNN, linear regression, LGBM, XGBoost, GBR, CatBoost, RF, and TTR based on the target variable’s case.

Before training the models and selecting the best-performing of them, the user has the right to intervene and define the optimal parameters of the model. Then the user switches to the next interface and specifies the bounds or the target value together with the monotony. This study adds to a dynamic optimization system in an actual Greek cement factory where the operator or the system defines the active artificial intelligence (AI) models and the expert determines the objective function. Essentially, the current method utilizes mathematical models to combine equipment optimization through mathematics and user participation to create a hybrid model. The machine operator’s experience should be combined with the AI model training and the optimization process. The expert is concerned with the data distribution and then utilizes the data clustering method. The clusters of data are divided based on the concept drift of the data. The occurrence of concept drift means that certain points of data signal a new distribution of data or a new behavior of data. In the next step, the intelligent decision making system (IDMS) interacts with the expert user. The user establishes the bounds of the non-manipulated parameters both for the kiln and the mill. The recommendations of the method are shown on the dashboard, where the user can determine whether they are appropriate values. Furthermore, the user possesses the ability to specify the parameters of the active prediction AI model. After training, the system can, nevertheless, choose the active AI model based on the *NRMSE* metric. Last but not least, the form of the objective function is defined by the user. In general, human–system interaction is the foundation of the entire system.

A main contribution of the paper is the reconciliation of non-manipulated variables with manipulated and uncontrolled variables using AI models. The AI models incorporate non-linear relationships between variables and changes in equipment condition and depreciation, accounting for periods of operation and non-operation. In order to ensure high accuracy, multiple AI methods are utilized to produce accurate forecasts. In this respect, the contribution of the user is also important, who triggers the models to be retrained when he/she considers that the behavior of the data has changed. An additional important contribution of the present work is that for the first time, a dynamic optimization system is created in a cement plant where the conditions and parameters that constitute the objective function are defined by the user. This property allows plant operators to define and compare different optimization strategies using various parameters. If the goal is to reduce the environmental impact, the objective function should consider both the mill’s environmental dust and the kiln’s fuel consumption. However, if the user considers that the material feed contributes to environmental impact, he/she may include it in the objective function with a weight of his/her choosing. The current project also contributes to the provision of a way to display factory data on the user interface and allows the user to select a specific time range. Additionally, the user can view the most recent data up to the last hour.

## 2. Related Work

More than half a century has passed since the first attempts to automate the process of cement production through the interconnection of the various parts of the factory with computer systems [19]. Many works have dealt with the optimal management of industrial units and cement plants. By exploiting exhaust gases [7] and utilizing high temperatures in the rotary kiln shell [20], thermal energy loss and the release of polluting gases are reduced. Nitrogen oxides in the kiln also contribute to the worsening of the environmental impact of cement industries [21,22]. The optimization of water, heat, and electricity was addressed in [23] and the optimization of nitrogen dioxide in [18], as well as in work [24]. The work [25] suggests that incorporating micro-encapsulated phase change materials (PCMs) into cement contributes to the reduction of energy consumption in buildings. In an extension of this work [26], a PCM based on decanoic acid/polyethylene glycol is created. The temperature of the lime kiln was investigated in [27]. However, individual parameter optimization does not create an overall reliable solution that optimizes the cement plant as a whole system. In work [4], several objective functions were created to reduce energy consumption, gas emission intensity, and economic cost. In the same work, nonlinear or linear relationships between parameters are not considered, thus, ML models could play this role. In work [28], the combination of AI and optimization methods is used to determine the optimal composition of the mix concerning the cement mortar materials. The study [29] considers the slurry’s optimal composition and the properties relevant to cement production. In the work [30], AI and optimization models are used to find the optimal point for environmental, economic, and engineering objectives in silica concrete production. The proportion of materials in concrete also occupied the work [31]. The ML models are created using nine (9) parameters. These models, in combination with heuristics, establish a system of proposals regarding the “recipe” of the concrete. The work [32] used ML models and an optimization algorithm to determine the content of free calcium oxide in cement clinker. In [33], a method for the management of big data coming from the installations of a cement plant is proposed. The analysis of the data and their presentation is carried out using an Enterprise resource planning (ERP) system. However, neither innovative optimization models nor AI models are involved in the process. The same idea is also the basis of the work [34], where more emphasis is placed on optimizing the presentation of the results in the user interface (UI) than on the data processing method. In this case, simple statistical methods are used. The work [35] proposes a method to optimize cost, energy, and material supply using PLC-based controllers. However, neither ML models nor an advanced mathematical optimization model are used.

The architecture and design of the mill and separator are altered to create an advanced process control (APC) system in work [36], which uses a costly method to improve the mill feed and cement fineness without allowing the user to provide feedback on the quality of the results in the control panel. In the current study, the user defines the optimization process via the dynamic dashboard. In [37], the problem in the optimal management of information systems in the cement industry is identified in terms of the system’s acquisition cost, usability, quick response time, and KPIs management. The work [38] aims to optimize the kiln and the quality of the clinker extracted from the kiln. Although AI models are being used and considerable data points are being taken into account, advanced optimization methods are not being employed, and the models are retrained with data of varying distributions. In the current study, these issues are solved. This work is an extension of the study [39], where now the objective function is dynamic, the parameters of the objective function have been enriched in both the mill and the kiln and the method has been integrated into a cement production process control system. Last but not least, in the current study, the user can determine the retraining of the models through the control table if he/she observes from the visualization of the data distribution based on the clustering method that there is a concept drift in the data. In our case, we have a small improvement in the performance of the algorithms after changing the data understanding in energy consumption. A comprehensive summary of the pertinent works is presented in Table 1, indicating the production process under consideration, inclusion of mathematical equations, and the corresponding method utilized.

## 3. Materials and Methods

### 3.1. System Overview

In general, the developed system is based on the production needs of the cement industry of Thessaloniki and aims to provide manufacturing industries with an autonomous, cognitive, intelligent, and reliable framework. Based on embedded mechanisms of collective cognitive thinking, the goal is to efficiently use and reuse raw resources and energy. Furthermore, the system aims to monitor, control, and optimize the production process performance while minimizing human intervention.

**Figure 1:**
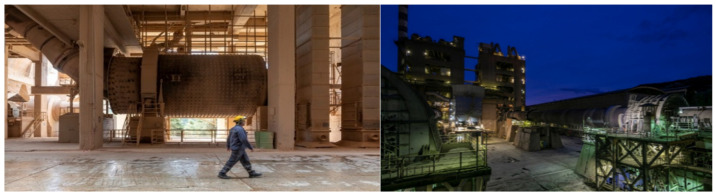
The cement industry facilities in Greece.

The system is a digital technology unit that utilizes an industrial Internet-of-Things (IoT) subsystem to collect real-time data from sensors during the production process. The system also employs algorithmic AI and decision-making tools to analyze the collected data, all to optimize the production process through predictive analysis. The industry facilities that interact with the system are demonstrated in Figure 1. The following are the basic functions performed in the cement mill and cement kiln.

#### 3.1.1. Cement Mill and Grinding System

A vertical mortar plant is utilized for grinding raw materials such as lime, clay, and other substances to produce cement. The operator can regulate several variables to control the process, such as:

Total Feed: This refers to the total amount of raw materials fed into the mill. The operator can adjust the total feed to control the residence time of the materials in the plant, which affects the fineness of the finished product.

Separator Speed: The separator plays a critical role in separating fine and coarse particles after grinding. By adjusting the separator speed, the operator can regulate the fineness of the cement. Typically, a higher separator speed leads to finer particles.

Fan Speed: The fan is responsible for circulating air in the mill. By adjusting the fan speed, the operator can manage the temperature and airflow within the mill, which influences the grinding process and the quality of the finished product (Blaine).

Water Flow: Water is usually sprayed onto the material inside the plant to manage the milling process and control the temperature. The operator can adjust the water flow to prevent overheating and regulate the consistency of the final product.

In terms of qualitative variables, Blaine is a measure of the cement particles’ fineness and strength. It can be controlled by adjusting the separator speed and total feed rate. On the other hand, residue refers to the amount of material left in the filter after grinding. This factor is influenced by the separator speed, total feed, and other variables. Operators must maintain the residue within certain limits to ensure the desired quality of the product.

#### 3.1.2. Cement Kiln

A kiln is a crucial component in the cement industry as it is used for producing clinker. The following description outlines the essential components and variables that an operator must manipulate to ensure the desired quality parameters in a kiln.

Kiln Feed: This refers to the raw materials, such as lime, clay, and other additives, that are fed into the kiln. The operator manages the kiln feed to control the clinker’s chemical composition and maintain the desired quality parameters.

Solid and Alternative Fuel Feed: Cement kilns can use alternative fuels like waste-derived fuels or biomass in addition to traditional solid fuels. The operator optimizes the feeds for combustion efficiency and maintains appropriate temperature profiles.

Kiln Speed: The rotational speed of the kiln influences the residence time of the materials inside. By adjusting the kiln’s speed, the operator influences heat transfer and chemical reactions, which affect the clinker’s quality.

Oxygen in the Preheater: Monitoring and controlling the oxygen level in the preheater is crucial for efficient combustion. Proper oxygen levels help achieve the desired clinker characteristics and prevent problems such as incomplete combustion.

Cooling Fan Flows: The fan flows in the cooler affect the clinker’s cooling process. By regulating these flows, the operator controls the cooling rate, which is critical to the final quality and characteristics of the cement.

Pressure Below the Grating: This parameter refers to the pressure below the cooler grate. Maintaining the correct pressure is crucial for proper operation of the cooling system and to prevent backflow of gases.

In terms of qualitative variables, free calcium oxide (Free CaO) refers to the amount of free calcium in the clinker, which is an essential quality parameter in clinker production. The free calcium levels can affect the cement’s properties and its behavior during storage and use.

### 3.2. System Architecture

The main responsibility of the physical layer–factory is to send sensory data to the system to optimize production. The exchange of this data is achieved by using message queuing telemetry transport (MQTT) protocol requests, which are received by the message broker. The message broker has direct communication with all the algorithmic tools/services in the system and, thus, forwards the data received to them.

**Figure 2:**
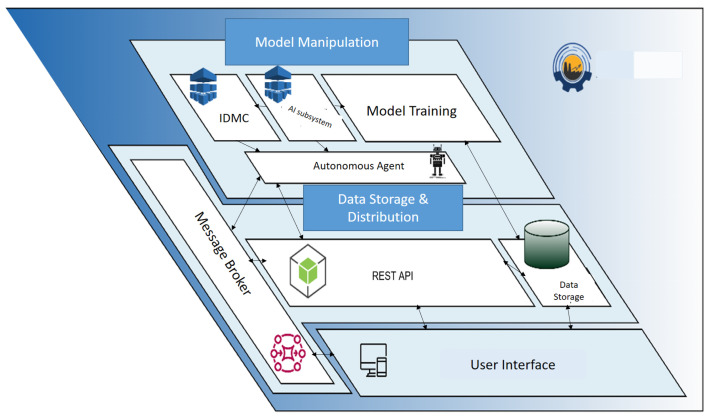
High-level architecture overview diagram.

The autonomous agent establishes connections between the individual algorithmic processes, namely, IDMC, model training, and AI subsystem, with the message broker and the REST API. Through the REST API, the user activates the autonomous agent in order to activate the algorithmic procedures. Additionally, the REST API interfaces with the message broker to provide notifications to the user regarding completed model training or recommendations from the IDMS. The information flow between the functions is shown in Figure 2.

The RESTful application programming interface (REST API) is the intermediary for any action to store or retrieve data from the database. This is how the algorithmic tools communicate: (i) with the message broker for data requiring real-time exchange and (ii) with the REST API to retrieve and/or store data in the database. The services of the system include the IDMS and AI subsystem tools, which exchange data between them through the message broker. These services utilize predictive models that are trained and acting during production. The message with the predictive data is also traced by the API, which stores the data in the database for future use. The autonomous agent tool is either a real user or an intelligent system. After obtaining the evaluation of the acting AI model (training parameters, performance metrics, historical data, historical predictions), the agent decides if the retraining routine should be called so the re-training parameters are entered into it.

### 3.3. System Sub-Modules

The sub-modules developed for the system consist of tools and technologies that are necessary for its operation. Using Docker allows the creation of images for each of these subunits and ensures that they work uniformly in every environment. The system sub-modules are listed below:

#### 3.3.1. The Mongo Database

The Mongo Database server sub-module is the one that hosts the database that is utilized by the system to store the data necessary for its operation. The Mongo database image includes the Mongo server and all the necessary dependencies for its operation. The Dockerfile for the database defines the sequence of actions to be performed to install and configure the Mongo database server inside the Docker container.

#### 3.3.2. User Interface (UI)

The graphical interface is one of the most important tools of the system, as it enables the user to manipulate the system functions. It is hosted by the Web Server, which includes all the necessary dependencies for executing the interface code and communicating it with the various sub-modules of the system. The interface, which is configured for the respective sub-module, is a central platform for collaboration between the different sub-modules of the system. It offers a set of methods and functions, allowing the other sub-modules of the system to exploit its methods for training, prediction, optimization, and decision-making in an automated and coordinated manner. Finally, the interface aims to ensure maximum flexibility and efficiency of the process by ensuring that the methods are extensively used by the other sub-modules of the system.

#### 3.3.3. Mosquitto MQTT Broker

The message broker sub-module is responsible for the real-time communication between the different sub-modules of the system. It includes all the necessary dependencies required for its operation and the security of the communication. Cedalo’s Mosquitto Management Center is a modern graphical environment that enables organized control and management of customers connected to an IoT architecture through the Mosquitto message broker, provided by Eclipse. It offers several features aimed at improving the process of managing and monitoring the information generated by customers. Some of these features include real-time data visualization, organizing customers into hierarchical groups, the ability to perform searches based on various criteria, and extracting data for further processing. Finally, Cedalo’s Mosquito Management Center offers easy configuration of security levels, allowing the definition of access rights for customers. The image for the message broker includes the Mosquitto message broker, an MQTT protocol server, and all the necessary dependencies for its operation. The Dockerfile for Mosquitto defines the commands to install and configure it.

#### 3.3.4. Mosquitto Management Center

The image of the Management Center includes its implementation and all the necessary dependencies for its operation. The Dockerfile defines the commands to be executed to install and start the management center application. The system’s services are divided into two main elements, as follows.

#### 3.3.5. AI Subsystem

The AI subsystem is responsible for applying the models generated by the retraining algorithm as well as generating predictions and making decisions. The AI subsystem combines the historical data with the recently collected data to provide real-time predictions of the values of the variables in the IDMS.

#### 3.3.6. Intelligent Decision Making System (IDMS)

The IDMS utilizes the methods provided by the AI subsystem and promotes the optimal values to the other sub-modules of the system. The DE optimization method is used to compensate the system for the optimized values of the manipulated variables, as derived from the combination of the optimization method of the AI methods and the feature selection methods.

#### 3.3.7. Receiving Real-Time Data

**Figure 3:**
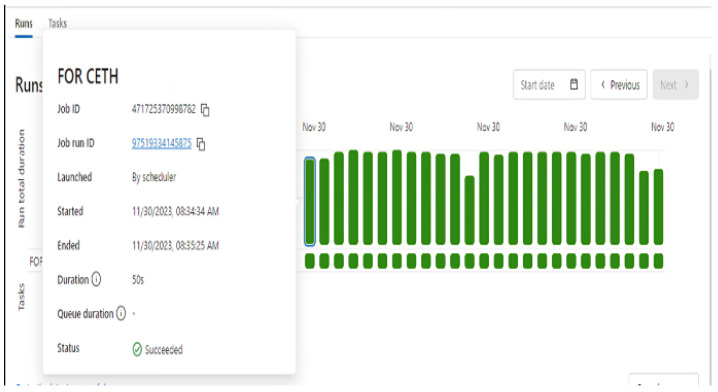
Monitoring the results of timed executions in the data reading algorithm.

The implementation of real-time communication between the system and the physical layer is based on cloud tools and services. Azure Databricks and Azure Data Lake are two critical tools for managing and processing large volumes of data. Data Lake is designed for data storage, supporting the ability to store data in various formats. The data are stored in the parquet format. This format is efficient in storing column-oriented data and provides high performance in reading and processing data. Azure Databricks is a powerful data processing and analysis tool. By leveraging Databricks, an algorithm for reading and sending data was developed using the Python programming language. In Figure 3, the Databricks interface is shown. The data are sent via HTTP request to the system’s server. The server receives the data from Databricks and verifies whether the received values exist or not. The new values received are stored in the system’s beta database, while those that exist are ignored. In this way, the data loss is minimized and duplicate values are avoided to achieve smooth and seamless real-time communication.

**Figure 4:**
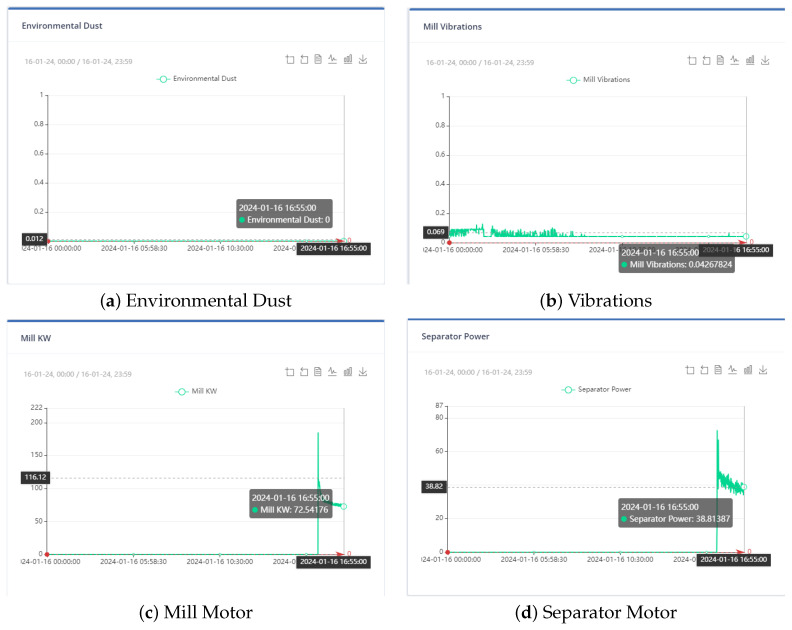

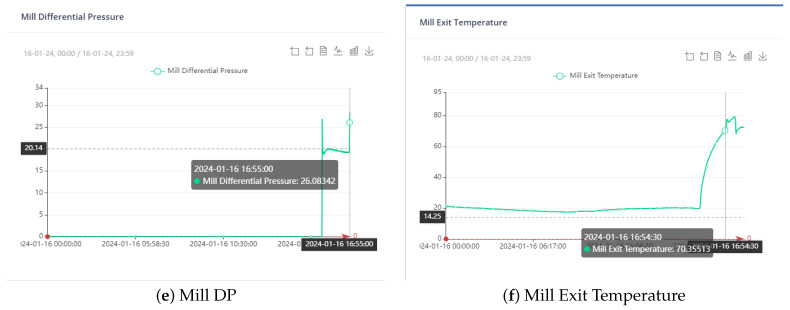
The distribution of data by non-manipulated variables in the mill and their value at a particular time point.

**Figure 5:**
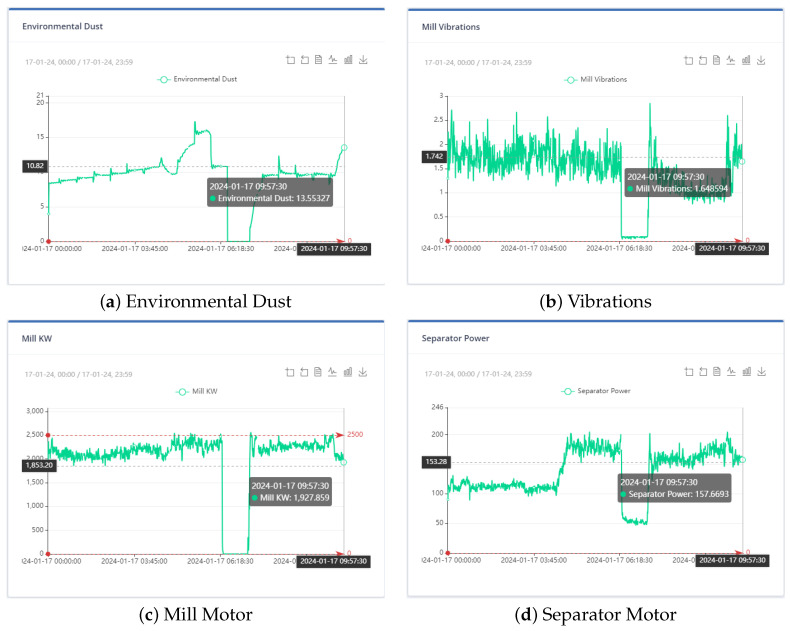

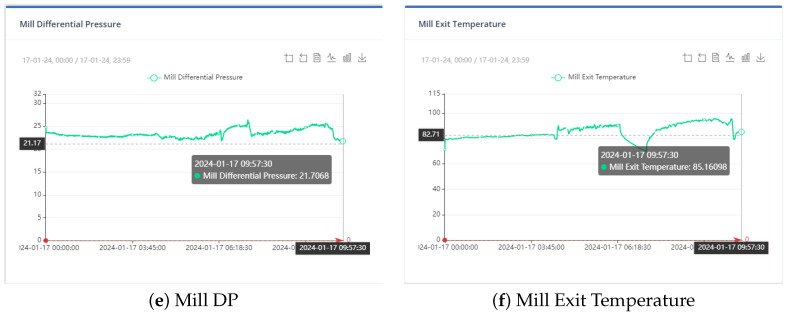
The distribution of data by non-manipulated variables in the mill and their value at a particular time point.

**Figure 6:**
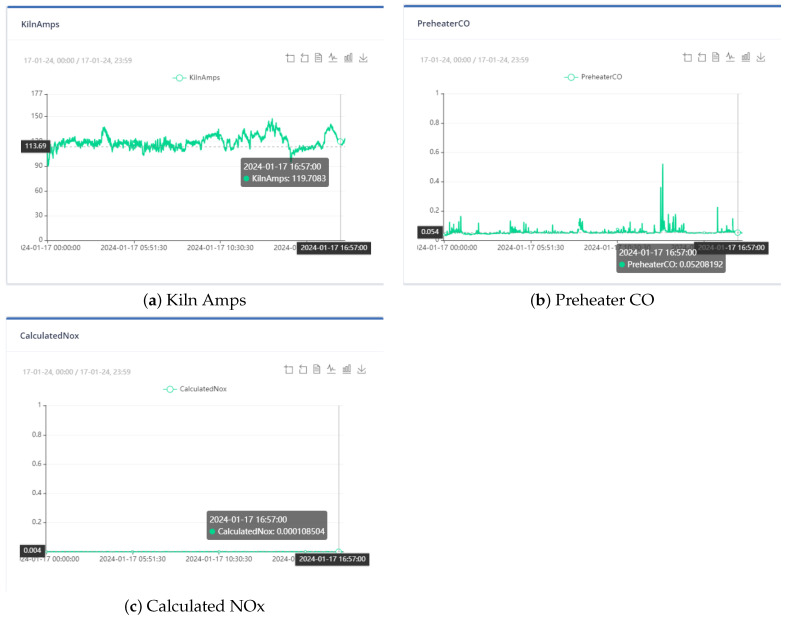
The distribution of data by non-manipulated variables in the kiln and their value at a particular time point.

Figure 4, Figure 5 and Figure 6 show the distribution of the mill and kiln data. Figure 4 and Figure 5 yielded the last data point in the mill optimization strategies, and Figure 6 yielded the final data point in the kiln optimization strategies. Based on the data distribution, the user gains an intuitive understanding of the presence of distinct data behavior and concept drift. The evolution of this system is the automatic detection of the concept drift of data and the subsequent activation of the AI training. In addition, the user has the possibility to export the data distribution in a predefined time range according to the options given in the UI, which are the following: Today, last twelve (12) hours, last six (6) hours, last two (2) hours, and customized width.

### 3.4. Data Description

#### 3.4.1. Cement Mill

The data used for the training of the initial AI models were from a period of almost three (3) years. Specifically, they consisted of the months of November 2020–December 2022 and June–July 2023. The data prepossessing involves the elimination of the data points where there is no mill operation. In the same context, the data relating to the first twenty minutes (20 min) after starting the mill operation were also discarded due to the instability of production in these intervals. The manipulated variables are the following: the mill feed, the grinding pressure, the separator speed, the water flow, the mill inlet pressure, the mill outlet pressure, and the mill inlet temperature. The prediction models are connected with the mill energy consumption, the mill differential pressure, the separator energy consumption, the mill exit temperature, the environmental dust, and the mill vibrations, as well as the cement fineness indices (Blaine and residue).

#### 3.4.2. Cement Kiln

The data used for the training of the initial AI models were obtained throughout approximately six (6) months. Specifically, they relate to the period 1 December 2022–12 June 2023. The first step in the data pre-processing process involved cutting out the intervals of kiln inactivity. Kiln operation was defined as those intervals during which the kiln feed rate exceeds 135 t/h. The manipulated variables are the total feed, the Preheater$O_{2}$, and the solid fuel feed. The kiln prediction models are associated with models that calculated nitrogen oxides, the kiln amps, and the preheater carbon dioxide.

### 3.5. Feature Selection Methods

For feature selection, we applied an automated selection procedure in which we combined two well-known methods: RFECV and SFS. This combined approach allowed us to identify a set of features that are highly relevant to the predicted variable. The subset resulting from the combination of the RFECV and SFS results is then introduced as input for training the prediction models.

#### 3.5.1. Recursive Feature Elimination with Cross-Validation (RFECV)

The features are initially given weights according to an estimator such as the coefficients of a model. In the RFECV method, features are eliminated iteratively by considering smaller sets of features in each iteration. The importance of each feature depends on some metric such as the pseudo-efficiency [40]. The least significant features are excluded from the selected features. The process is terminated when the desired features reach the desired number. The RFECV method additionally uses the stratified validation method in the selection of mapping features [41]. In our case, the implementation of the method was based on an estimator that consists of a serial procedure created with the sklearn.Pipeline python library. The transformation of the data includes normalization with the min–max method and training with the linear regression method. The measurement of the transformation is the negative root mean squared error.

#### 3.5.2. Sequential Feature Selector (SFS)

This method is based on the greedy approach, where at each step, a feature is added or removed based on the overlap method [42]. However, it only considers the independent variables in the training process and not the dependent variable to be predicted [43]. More specifically, in this approach, both the method estimator and the performance measure are carefully chosen. The creation of a sequential series of data transformations is facilitated by the sklearn.Pipeline library of the Python programming language. Once the data is normalized using min–max normalization, the assessment of the model’s performance is carried out by examining each feature individually based on linear regression. The approach involves commencing with a singular attribute and evaluating its performance using k-fold cross-validation, as well as the negative root mean squared error metric. The metric is derived by calculating the negative distance between the estimated and actual data values [44]. With each iteration, significant attributes are introduced until the performance remains below the predefined value defined by the tol variable, which equals $10^{-3}$. The forward approach is employed as the direction parameter is set to forward.

### 3.6. Artificial Intelligence Methods

The system selects by itself the optimal parameters for each model with the GidSearchCV library. The selection of the best model is based on the *NRMSE* metric. In the absence of user intervention, the model chosen is the one that combines the algorithm and parameters with the lowest *NRMSE* for each non-manipulated variable. In rare instances, the user may possess knowledge contrary to their typical experience regarding the associations between independent variables (manipulated variables) and dependent variables (non-manipulated variables).

As an illustration, it can be observed that a non-manipulated variable prediction model does not include any manipulated variable. In this scenario, the user has the option to develop a model that incorporates one or more manipulated variables alongside the selected features. In these extraordinary instances, the user has the option to manually choose the active model. Randomly generating an active model can potentially disrupt the optimization system and produce recommendations that are deemed unacceptable. As a result, the user’s decision to manually train a model is a matter of utmost importance and is undertaken only in exceptional instances. Subsequently, the model’s efficacy can be assessed based on the outcomes it generates within the optimization system. Below are the implementation details for each AI model.

#### 3.6.1. Multi-Layer Perceptron Regressor (MLP)

By calculating the partial loss derivatives at each MLP regressor training cycle, the parameters are updated. In the loss function, the model parameters are reshaped using a regularization term to avoid overfitting [45]. For the MLP model, the best value for the hyperparameter hidden layer sizes was chosen among (1,), (2,), (5,), (1,1), (2,2), and (5,5), where the number of hidden layers and the number of neurons in each is determined. Furthermore, tanh was set as the activation function, blogs as the optimization method (solver), 108 as the maximum number of iterations, and the regularization parameter α was set equal to 0, as these options seemed to give generally better results than the default values of these hyperparameters, based on preliminary experiments.

#### 3.6.2. Gradient Boosting Regressor (GB)

By creating a progressively additive model through an estimator, it is possible to optimize random differentiable loss functions. The negative slope of any given loss function is mapped to a regression tree [46]. For the GBR model, the best value for the max depth hyperparameter was chosen between the values 5, 10, and 15 for the number of estimators hyperparameter among the values 50 and 100, while for the min_samples_split hyperparameter, the value 8 was set instead of the default, which is 2, as it seemed to give better results based on preliminary experiments.

#### 3.6.3. Light Gradient Boosting Regressor (LGBM)

LightGBM is a type of GB Regressor where discrete buckets sort continuous values based on histogram algorithms. Thus, it speeds up the training process while being a computer-memory-friendly process [47]. For the LGBM model, the best value for the max depth hyperparameter was chosen among the values 5, 10, and 15 and for the number of leaves hyperparameter among the values 8, 10, and 12.

#### 3.6.4. Extreme Gradient Boosting Regressor (XGBoost)

The XG Boost algorithm is a variant of the GB algorithm and is based on trees to create regression models using gradient boosting. The advantages of the XG Boost algorithm are summarized in the intelligent splitting of trees into short leaf nodes and randomization [48].

#### 3.6.5. Random Forest (RF) Regressor

A large number of decision trees create a meta-evaluator RF. Each of the decision trees is mapped to different subsets of the dataset. The average error improves the predictive accuracy while controlling for overfitting. The size of each subset of the sample is expressed by the hyperparameter max; otherwise, each tree is generated from the entire dataset [49]. For the RF regressor model, the best value for the hyperparameter number of estimators was chosen among the values 10, 15, 20, 25, 50, and 100 and for the hyperparameter max depth among the values 1, 2, 5, and 10.

#### 3.6.6. K-Nearest Neighbors (KNN) Regressor

In this method, uniform weights are used. That is, in each local neighborhood of a point, the distribution of candidate solutions is uniform. Depending on the case, weights are given to nearby points or distant points [50]. For the KNN, the best value for the hyperparameter number of neighbors was chosen among the values 5, 10, 50, 100, and 200 and for the hyperparameter leaf size among the values 20, 30, and 50 and the hyperparameter weights between the values of uniform and distance.

#### 3.6.7. Linear Regressor

In a linear regression analysis, there is the dependent variable to be predicted and the independent variable that is known. The model is essentially a linear correlation between the dependent and independent variable or independent variables [51].

#### 3.6.8. Cat Boost Regressor

Combining decision trees with the greedy approach and gradient boosting theory to combine several weak models at each step of the algorithm is the main idea behind the CatBoost method. Each new tree improves its performance by learning from the errors of previous trees until the intercept of the performance metric is not reduced [52].

#### 3.6.9. Transformed Target Regressor (TTR)

This is used for non-linear transformations of the dependent variable. Either a transformer such as the quantile transformer or its inverse is used [53]. For the CatBoost model and the TTR model, their hyperparameters were set equal to their default values.

### 3.7. Clustering Data Method

#### K-Means

The k-means algorithm is an unsupervised ML algorithm that clusters data points that have some similarity. The number of clusters is predefined and the method sets the center of each cluster (centroid). This value is representative of each cluster [54]. Depending on the distance from each point to the centroids, the algorithm assigns it to the least distant cluster.

In order to accommodate the variable to be grouped, such as the total feed, the k-means algorithm sets two behavior groups. The variable data would have been normalized with the standard scaler in previous steps. Once the data points have been divided into two groups, the resulting data from the dabricks are also divided into two groups. This division is based on a threshold that is equal to the average value obtained from the maximum value of the variable in the first group and the minimum value of the variable in the second group, or vice versa.

### 3.8. Evaluation Measures

#### 3.8.1. Normalized Root Mean Squared Error (*NRMSE*)

The *NRMSE* is a variant of the *RMSE*. The *RMSE* is calculated according to Equation (1). The smaller the *RMSE*, the closer the predictions are to the actual prices.

(1) $$RMSE=\sqrt{\frac{\sum_{i=1}^{n}{\left(y_{i}-\hat{y}\right)}^{2}}{n}}$$

where $y_{i}$ is the actual value of variable and $\hat{y}$ its predicted value. The n reflects the number of observations. The values that have a different measurement from the active forecasts cannot be compared, and therefore, the construction of a metric that can be applied to all variables is necessary [55]. This metric is the *NRMSE*, which weights the mean squared error in terms of mean, standard deviation, difference between maximum and minimum, or interquartile range. When the *NRMSE* is equal to 0 it means that the predictions coincide with the actual values [56]. In the current study, the mean square error is normalized with respect to the standard deviation. The normalized form of the mean squared error is mainly useful in large statistical samples and in cases of heterogeneous outcome variables. The *NRMSE* is calculated according to Equation (2). The std(y) is the standard deviation of the variable’s distribution.

(2) $$\begin{array}{c} NRMSE=\frac{RMSE}{std(y)} \end{array}$$

#### 3.8.2. Quantitative Change

This metric measures how well production is optimized. It is calculated by comparing the active value with the optimum value of the objective function from the DE. The active value of the objective function is obtained by substituting the values of the most recent data point in the user-defined objective function. Similarly, the optimal value of the objective function is obtained by substituting the values of the manipulated variables proposed by the DE into the objective function. The Equation (3) illustrates how to calculate the quantitative variation.

(3) $$\begin{array}{c} QC=\frac{F_{optimization}}{F_{LastDatapoint}}-1 \end{array}$$

### 3.9. Objective Function

The objective function is also the KPI of the application. Essentially the degree of optimization of the cement production process is user-oriented. The user dynamically determines the form of the key performance indicator, which is equal to the objective function as Equation (4).

(4) $$\begin{array}{c} F=\sum_{n=0}^{N}[W_{bounds_{n}}\times [W_{min_{boolean}}\times {\left(\frac{var_{n}-minimum_{n}}{std_{n}}\right)}^{2}\times (boolean\left(var_{n}<minimum)\right)+W_{max_{boolean}}\times \\ {\left(\frac{var_{n}-maximum_{n}}{std_{n}}\right)}^{2}\times \left(boolean\left(var_{n}>maximum\right))\right]+sign_{n}\times W_{monotony_{n}}\times \frac{var_{n}-mean_{n}}{std_{n}}+W_{target_{n}}\times {\left(\frac{var_{n}-target_{n}}{std_{n}}\right)}^{2}]\; \end{array}$$

where $W_{bounds_{n}}$ is the weight the user assigns to the variable when it is selected as the variable to be within constraints. The $W_{min_{boolean}}$ and the $W_{max_{boolean}}$ are equal to either the value 0 or 1 depending on whether the upper and lower ends of the constraints are selected, respectively. The $boolean(var_{n}<minimum)$ and $boolean(var_{n}>maximum)$ are both Boolean variables that are equal to *False* until the active value of the variable is either less than the minimum threshold (*minimum*) or greater than the maximum threshold (*maximum*). These thresholds are set by the user. $sign_{n}$ is a binary variable where it is equal to −1 if the user chooses to maximize the current variable and 1 if the user chooses to maximize the current variable, since DE minimizes the objective function. $W_{monotony_{n}}$ equals the weight the user gives to the monotony of the corresponding term in the objective function. $W_{target_{n}}$ is the weight given if we choose for the variable to have values close to a target value. In case we choose a target value, both the choice of bounds and monotony are disabled. $target_{n}$ expresses the target value, $var_{n}$ expresses the actual value of the variable at the current step in the optimization process, $std_{n}$ expresses the standard deviation, and finally, $mean_{n}$ expresses the mean value of the variable from the data.

If the selected variable $var_{n}$ belongs to manipulated variables, it receives the current value within the predefined bounds (value(n)), and if the variable is non-manipulated, then the value of the variable depends on the linear or non-linear relationships between the selected variables. These variables were determined by the feature selection algorithms, while the dependent value is estimated by the active ML models (prediction(n)), as is represented in Equation (5).

(5) $$var_{n}=\left\{\begin{array}{cc} prediction(n) & \mathrm{if}\;n\;\mathrm{in}\;\mathrm{non}\text{-}\mathrm{manipulated}\;\mathrm{variables}\\ value(n) & \mathrm{if}\;n\;\mathrm{in}\;\mathrm{manipulated}\;\mathrm{variables} \end{array}\right.$$

### 3.10. Differential Evolution (DE)

The DE is a metaheuristic of continuous iterations until the candidate solution is judged to be the best. The initial assumptions about the problem are few or none and the space of candidate solutions is very large. In general, DE is considered a stable and reliable optimization algorithm. The origination of the method is in work [57]. The studies [58,59,60,61] prove that DE is the most stable and reliable optimization algorithm. The DE method belongs to the stochastic evolutionary methods, chooses the best among numerous candidate solutions, and has only three (3) parameters. The existence of a few parameters is one of its main advantages. The main disadvantage of the method is that the selection of the best parameter values is a hard task. Finding the optimal values of the control parameters can be time-consuming and difficult, especially for difficult problems. For these reasons, researchers study and develop new advanced DE variants that exhibit adaptive or self-adaptive control parameters. The control parameters are adjusted based on the feedback received during the search process [62]. In our case, the bound intervals of the manipulated variables are the range between thirty percent (30%) less and thirty percent (30%) more than the corresponding value of the last data point. We set the maximum number of iterations to three hundred (300), which is quite a high number, while the population size of candidate solutions is equal to five (5). The number of iterations is high since the problem is likely to be multidimensional and the value of iterations depends on the ability of the algorithm to find candidate solutions [63]. The optimization method is represented in the Equation (6).

(6) $$\min_{x_{i}}\left(\sum_{i=1}^{N_{e}}L_{i}\right)\;s.t.\;low\;bound\leq g_{k}\left(x\right)\leq \;up\;bound,\;k=1,\ldots ,K$$

where $N_{e}$ is the number of parameters, *x* = {$x_{1}$, …, $X_{N_{e}}$} is the vector containing the candidate solution areas $x_{i}$ of all elements, and $L_{i}$ is the value of element *i*. In addition, $g_{k}\left(x\right)$ is the behavioral constraint of K variables. At each step, the algorithm generates a new candidate solution by combining the existing solutions of the previous step. The candidate solutions give the objective function the best value, which is either the highest or lowest according to monotony, at each step. The procedure is iterative until the satisfaction of a predefined criterion [64].

### 3.11. Flowchart of the Platform

At the outset, the user can examine the data distribution of the manipulated variables through the user interface, illustrated in Figure 4, Figure 5 and Figure 6, to determine any potential variations in behavior. In the event that the data exhibits concept-drift behavior, it is necessary to inform the developer to retrain the models using two subsets of data. It is important to note that a significant limitation of the system is its inability to automatically detect concept drift in the data, requiring human intervention. Subsequently, the user is directed to the interface for AI model training. Under extraordinary circumstances, the user determines the model to be trained; otherwise, the system automatically renews the active ML models by extracting the most suitable parameters.

**Figure 7:**
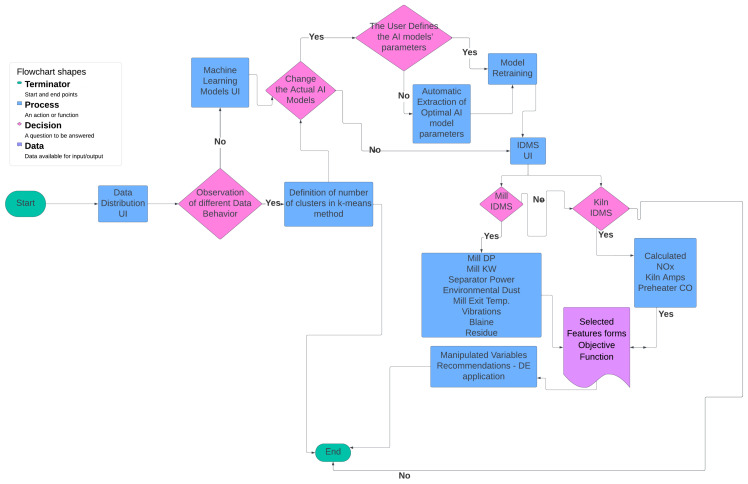
The flowchart of the platform.

Following the retraining and exporting process of the best-performing models, which are also the active models unless the user manually trains them, the user navigates to the IDMS interface. Within this interface, the user has the ability to determine the parameters of the linear objective function by selecting the non-manipulated variables that will be included. Once the user has defined the parameters via the user interface, the system generates suggested values for the manipulated variables on the screen, as long as the optimization is considered appropriate. The described flowchart is shown in Figure 7.

The analysis of the AI subsystem should be approached as a process that is either dependent on the user or the algorithm. Initially, the user chooses the forecast model, either for the mill or the kiln in this particular scenario. Subsequently, the user establishes the dependent variable on which the training is conducted. This variable is predicted by the output model after the training process and should be a non-manipulated variable. The user or the RFECV and SFS feature selection algorithms are responsible for selecting the features to be trained in the first scenario and in the second scenario, respectively.

**Figure 8:**
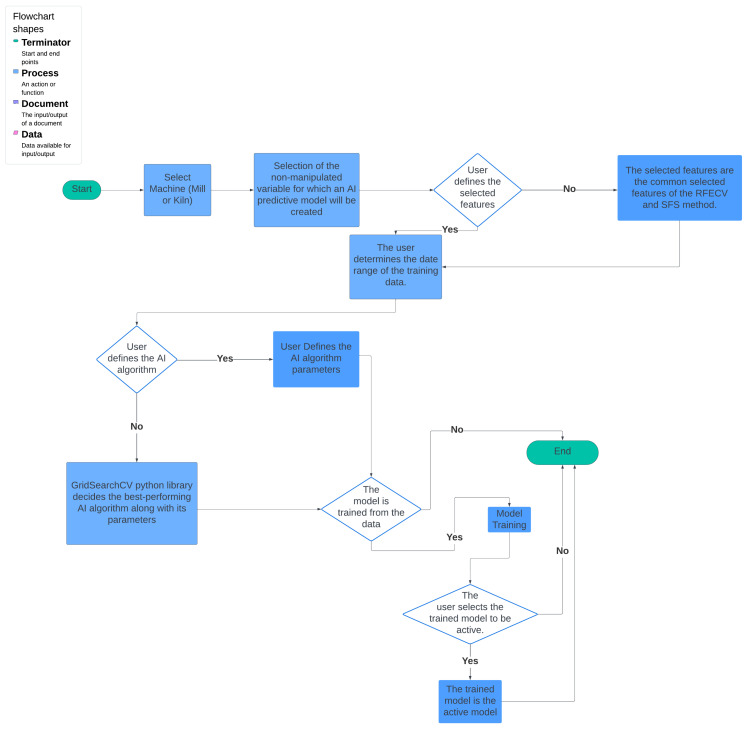
The flowchart of the AI subsystem.

Upon reaching the subsequent interface, the user is presented with the option to either personally determine the training model and its parameters or delegate the task to the system, which selects the most suitable AI model and its parameters. When considering the first scenario, it is imperative that the user possesses a profound understanding of the problem in order to establish these parameters. The upcoming interface determines whether the user sets the model as active or saves the parameters for future use without training the model. If the user does not take any action, the system determines the best model according to the *NRMSE* measure. The whole AI subsystem flowchart is depicted in Figure 8.

The ultimate goal of the system is to create recommendations for optimal operation to the user through the IDMS system. In this case, the user chooses which parameters—manipulated or non-manipulated variables—participate as terms in the objective function (4) or receive a weight equal to 0 so they do not participate in the objective function. Depending on which parameters are selected by the user interface, the objective function optimizes a specific aspect of the cement plant, such as product cost, environmental footprint, or energy consumption.

**Figure 9:**
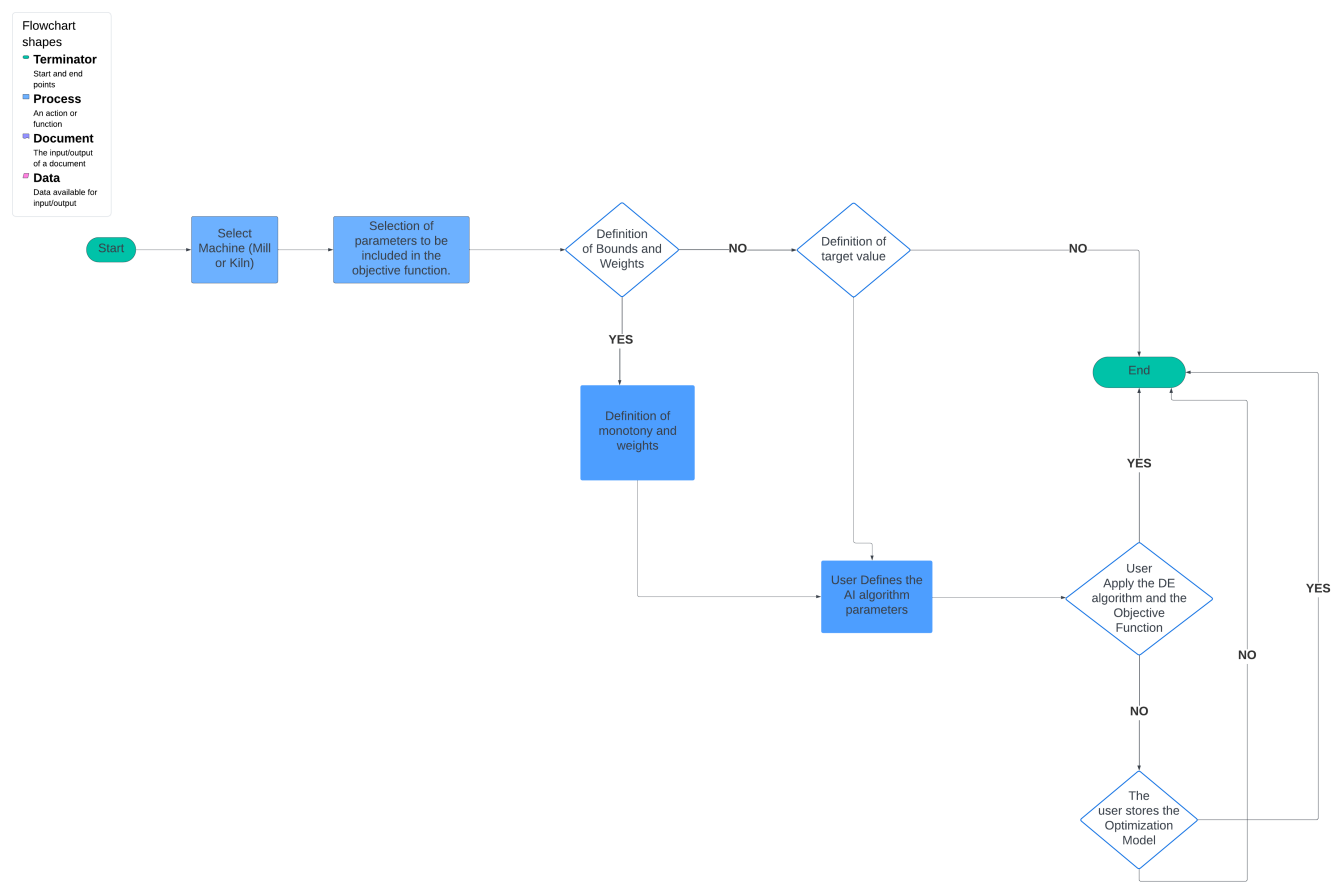
The flowchart of the IDMS subsystem.

The variables to be optimized are determined either within a predetermined interval or around a predetermined target value, depending on the case. In the initial scenario, it is crucial to establish the monotony of the variable, that is, whether its objective is to be minimized or maximized. In the second instance, the algorithm works towards reducing the distance between the variable and the desired value. For the case described, there is the corresponding user interface where the aforementioned parameters are defined. In the following user interface, the parameters of the DE method such as the number of iterations and the population size are defined. The information flow of the IDMS subsystem is illustrated in Figure 9.

## 4. Results

### 4.1. Data Clustering

**Figure 10:**
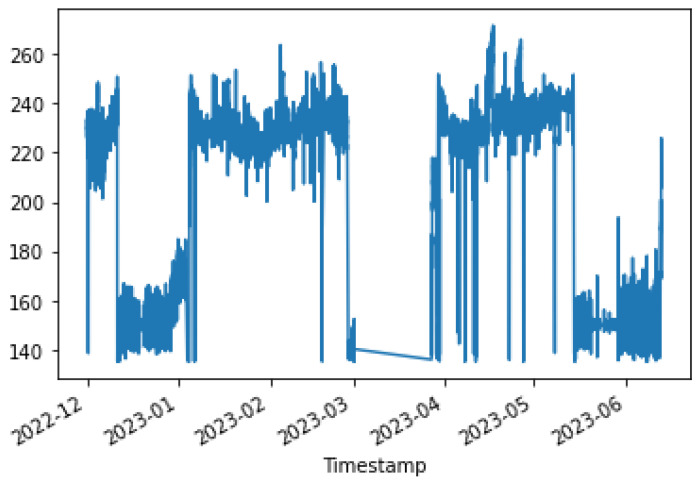
The Distribution of the Kiln Feed parameter.

**Figure 11:**
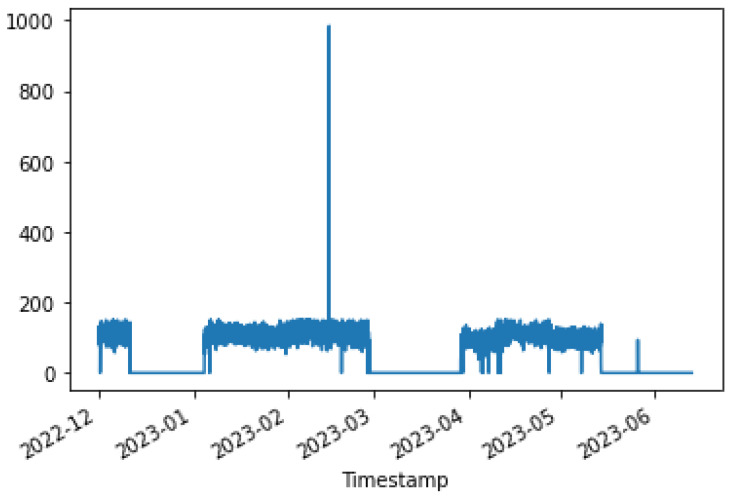
The Distribution of the Kiln Amps parameter.

The data in the kiln feed exhibit two distinct distributions over time. As can be observed from Figure 10, the first distribution describes the feed between 140 and 160 tons, while the other describes the feed between 220 and 240 tons. The Kiln ambers distribution is drawn in Figure 11. Kiln ambers are almost zero (0) during the lowest kiln feed period, which is observed to indicate that the kiln is not operational. The model that is created predicts the non-operational periods even when the kiln loading exceeds 135 t/h.

### 4.2. AI Subsystem Results

Feature selection methods RFECV and SFS seek the features of the data that create the best-performing model. The intersection of the results of the two methods, i.e., the common features, are those with which the ML models were trained. These characteristics are represented in Table 2 for the mill and in Table 3 for the kiln. Given that there are only three (3) manipulated variables in the kiln, the selected features consist of the combined set of social features identified by the methods and the manipulated variables.

During the training of ML models, the one that performs best is always selected. This is not an easy process, as the performance of the models often depends on the hyperparameters of the algorithms and their numerous combinations. Therefore, it is necessary to find a way to tune the hyperparameters to the set of hyperparameters of the best-performing model [63]. The Python library scikit-learn with the GridSearchCV method provides a hyperparameter tuning tool in an automatic and fast way [65].

Table 4 and Table 5 represent the results obtained in each step for the cement mill and kiln, respectively. After training the AI regression models, the represented results show the best-performing model for each variable, as well as its test *NRMSE* and its optimal parameters.

The results in Table 6 and in Table 7 are derived after applying the feature selection methods combined with the clustering method for the cement mill and kiln, respectively. Indeed, the clustering method achieves the segregation of data into operational periods. The *NRMSE* of the models is examined in the case where there are two behaviors in the data. The total *NRMSE* is obtained by summing the squared errors of all classes in a single square root in the numerator, which is equal to the deviations between the actual value and forecast and the denominator, which is equal to deviations between the actual value and the mean value.

Observing Table 5 and Table 7, the results imply that in the kiln, although there are two different behaviors in the kiln feed and in the kiln amps, the model retraining brings no improvement in *NRMSE* and, in all cases, marginally worsens it. For the case of the mill (Table 4 and Table 6), the results in some variables are improved and more specifically for the non-manipulated variables mill differential pressure, environmental dust, Blaine, and residue; however, the improvements in RNMSE are again marginal.

### 4.3. Cement Mill Optimization Results

#### 4.3.1. Experimental Set Up and Optimization Strategies

In our particular scenario, four distinct optimization strategies are implemented to enhance the mill’s performance. The initial approach incorporates all non-manipulated variables, along with the mill’s feed, into the objective function using weighted grading. The second optimization strategy solely utilizes the mill feed, along with the mill and separator energy consumption. The objective of the second strategy is to achieve low power operation in the mill without considering any other factors. The third optimization strategy reflects the environmental impact of the mill production and the fourth strategy reflects the production cost.

**Figure 12:**
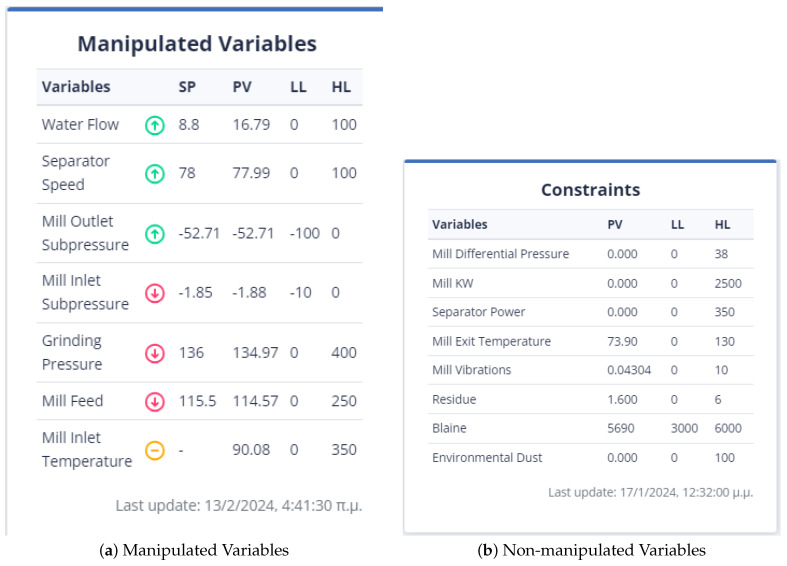
The constraints, the process values (PV), the setting points (SP), and the range of constraints (LL, HL) if a manipulated or non-manipulated variable is close to zero (0). The green colour arrow means that the PV is increasing, the red colour arrow that it is decreasing and the yellow colour arrow that it is stable.

To ensure that the optimization results are applicable to the cement plant, the bounds for both non-manipulated and manipulated variables are set to differ by a range of ten to thirty (10–30) percent from the value of the variable in the last data point. If a manipulated or non-manipulated variable is close to zero (0), it is constrained to the limits set in the user interface, which are shown in Figure 12. The last recorded data points were on 13 December 2023 10:50:00 PM, 16 January 2024 16:55:00, 17 January 2024 09:57:30, and 17 January 2024 10:45:30 for the first, second, third, and fourth strategies, respectively. The Figure 4 and Figure 5 display the values of the manipulated variables for the last data point of the second and third optimization strategies, respectively. The initial optimization strategy assigns a weight of one (1) to the mill feed, which is the smallest, as the impact of the mill feed on other optimization parameters is expected to be minimal. The second optimization strategy involves assigning a weight of $10^{3}$ based on the cement plant’s suggestions that reducing energy consumption is connected to increasing mill feed. The fourth strategy assigns a weight of $10^{2}$. In this point, the current study differs from the previous one [40] where the mill feed was the most important variable. The weight given to these parameters is equal to 10 for the first strategy. The second as well as third strategies do not concern cement fineness. The fourth strategy gives higher weights on these factors than the first strategy as the cement quality parameters affect product cost. The Blaine index obtains weights equal to $10^{3}$ and the residue index obtains weights equal to $10^{2}$ in the fourth strategy.

The mill differential pressure, the environmental dust, and the mill exit temperature are parameters added to this work. For the first optimization strategy, the weight to be given is equal to $10^{4}$, and it is desirable to be maximized. One main goal is the improvement of the environmental footprint, so the environmental dust should be minimized. The weight given is equal to 100 for the first optimization strategy and $10^{5}$ for the third one. The mill exit temperature should be maximized to save energy. The weight assigned to the variable is equal to $10^{3}$. The second optimization strategy does not include these parameters as it wants to put all the emphasis on energy saving. The third optimization strategy assigns high weight on environmental dust, equal to $10^{5}$. Last but not least, the fourth optimization strategy does not take into account these factors.

The power consumption of the mill and separator and the vibrations of the mill are the most important variables in the optimization process for both optimization strategies. For this reason, they are given weights of $10^{6}$, $10^{5}$, and $10^{6}$, respectively, for the first optimization strategy. The second optimization strategy assigns no weight to vibrations but assigns a significantly high weight ($10^{6}$) to the energy consumption of the mill machine and a high weight ($10^{5}$) to the energy consumption of the separator. The same weights for the power consumption factors are in both the third and fourth optimization strategies. Table 8 represents the weights of each factor and the main point of each optimization strategy. In each bracket in the table next to the weights, we indicate whether it is desirable for that variable to increase or decrease.

The constraints of the manipulated variables were specified in the source code. The input for the DE method includes the constraints of the manipulated variables. The source code incorporates dynamic form and includes constraints on manipulated variables that are within a range of 30% above and below the last data point.

#### 4.3.2. Setting Parameters from the UI

**Figure 13:**
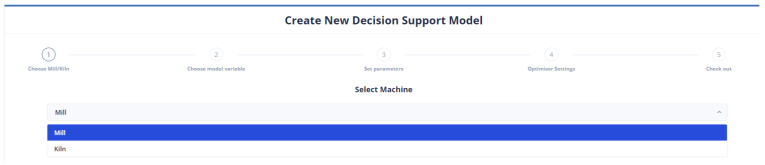
The option between mill and kiln in the dashboard to determine the corresponding objective function.

Figure 13 demonstrates the user’s initial selection in the control panel. The optimization applies to either the mill or the kiln. Of course, the user has the right to undo his selection and return to this step.

**Figure 14:**
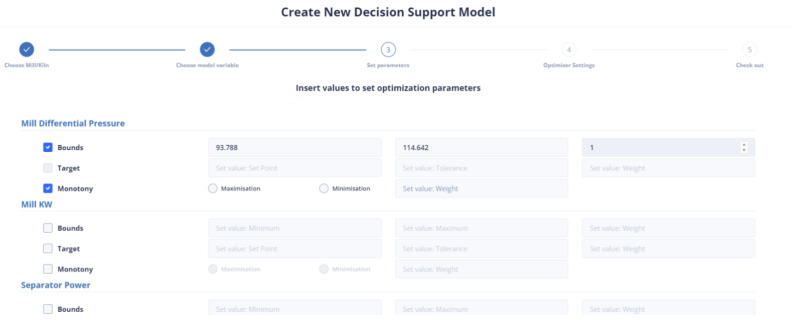
Creation of Objective Function via the Dashboard.

Figure 14 represents the user’s ability to specify the parameters that are included in the objective function. If the user does not select any parameter, then the weight to be given to it is set equal to zero (0). The user has the right to select the constraints of the variable together with its monotony or a target value for this variable. In the case where the constraints are chosen, he/she may not choose a target value and vice versa. Furthermore, if the target variable is selected, the user cannot specify the monotony.

- First Optimization Strategy

**Figure 15:**
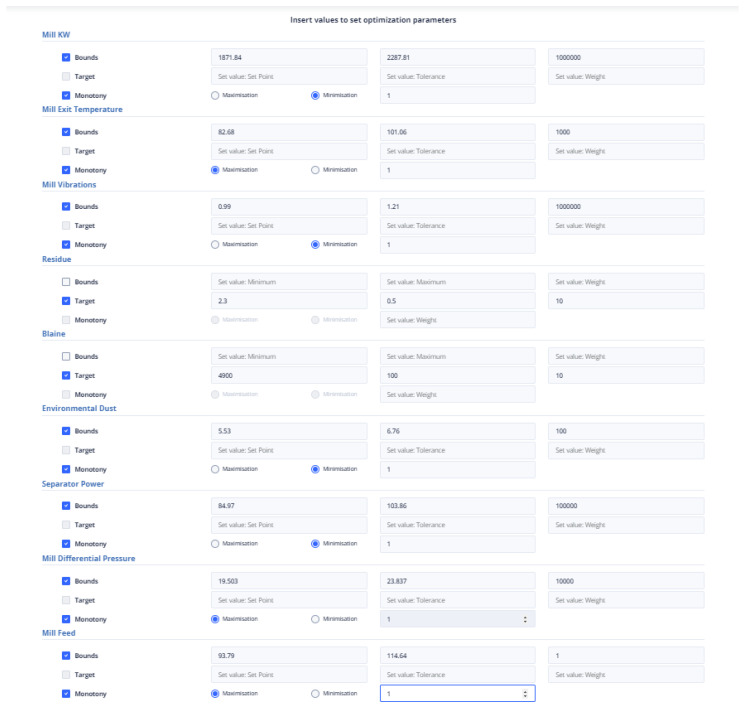
Creation of Objective Function of the 1st Optimization Strategy via the Dashboard.

The mill feed value in the latest data point is 104.2245. This means that the range of constraint is between 93.79 and 114.64. The first strategy being used in the current study is similar to the approach used in a previous study [40]. In this strategy, target values were set for the Blaine and residue indices, which are 4900 and 2.3, respectively, and their tolerance was set to 100 and 0.5, respectively. Additionally, the most recent reading for the mill differential pressure is 21.67, and the optimal value is expected to lie within the range of 19.503 to 23.837. As for the environmental dust, the last recorded value is 6.14, and the optimal value is anticipated to be between 5.53 and 6.76. The mill exit temperature was recorded at 91.87, which is outside the optimal range of 82.68 to 101.06. Additionally, the mill power consumption was 2079.82 KW, the separator power consumption was 94.421 KW, and the mill vibration was measured at 1.102364 mm/s. To optimize these values, the following intervals need to be considered: [1871.84, 2287.81] for mill power consumption, [84.97, 103.86] for separator power consumption, and [0.99, 1.21] for mill vibration. Figure 15 represents the introduction of constraints, target-values, monotony, and tolerance of non-manipulated or manipulated variables in the first optimization strategy.

- Second Optimization Strategy

**Figure 16:**
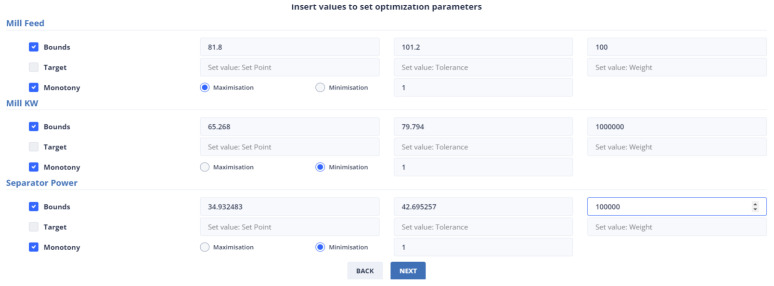
Creation of Objective Function of the 2nd Optimization Strategy via the Dashboard.

At the last data point we used for the second strategy, the energy consumption from the mill was 72.54 KW and the energy consumption from the separator with 38.81 KW. The mill feed was equal to 101.2 tons. The intervals within which the optimum values are found are [65.268, 79.794], [34.932, 42.695], [81.8, 101.2] for the variables energy consumption from the mill, energy consumption from the separator, and energy consumption from the separator and mill feed, respectively, and as shown in Figure 16. The weights to be assigned to these variables are shown in Table 8.

- Third Optimization Strategy

**Figure 17:**
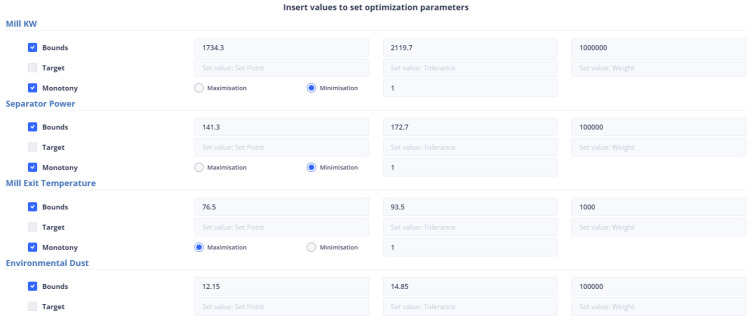
Creation of Objective Function of the 3rd Optimization Strategy via the Dashboard.

In the third optimization strategy, the values of the non-manipulated variables were as follows: environmental dust equal to 13.55, mill vibration equal to 1.64, energy consumption in the mill equal to 1927.85, energy consumption in the separator equal to 157.66, differential pressure equal to 21.7, and exit temperature in the mill equal to 81.5. The boundaries of the constraints within which the optimum value is found are 10% above and below the current value, as in Figure 17. The weights assigned to the variables were shown in Table 8.

- Fourth Optimization Strategy

**Figure 18:**
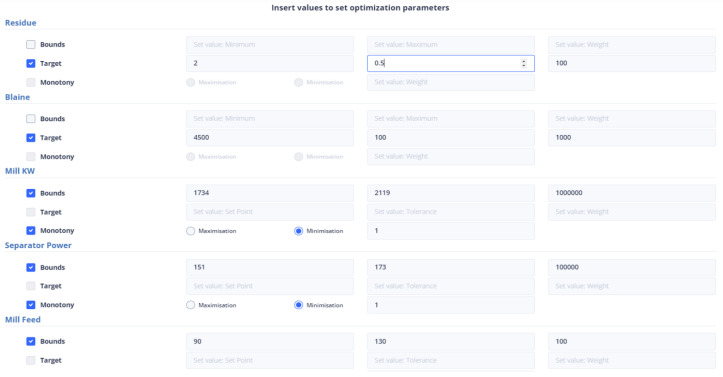
Creation of Objective Function of the 4th Optimization Strategy via the Dashboard.

This is the second strategy that utilizes the cement fineness indices Blaine and residue, in addition to the first one. In this approach, the target values are specified, along with a tolerance for deviation from these values, as is depicted in Figure 18. The final data point taken into account is the same as the one considered in the third optimization strategy.

**Figure 19:**
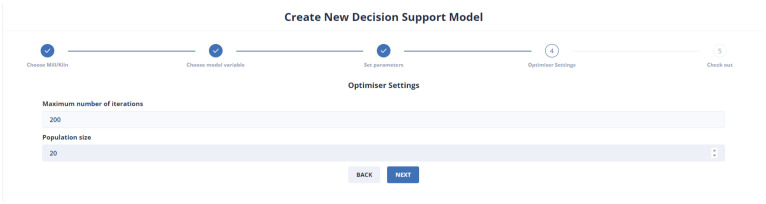
Determination of the DE optimization matrix parameters via the Control Panel.

Finally, the user selects the parameters of the DE optimization method, as is represented in Figure 19. These parameters are important in the efficiency of the method. More specifically, the population size determines the initial population, while the number of iterations is the upper limit of steps of the algorithm.

#### 4.3.3. Cement Mill Results

- First Optimization Strategy

**Figure 20:**
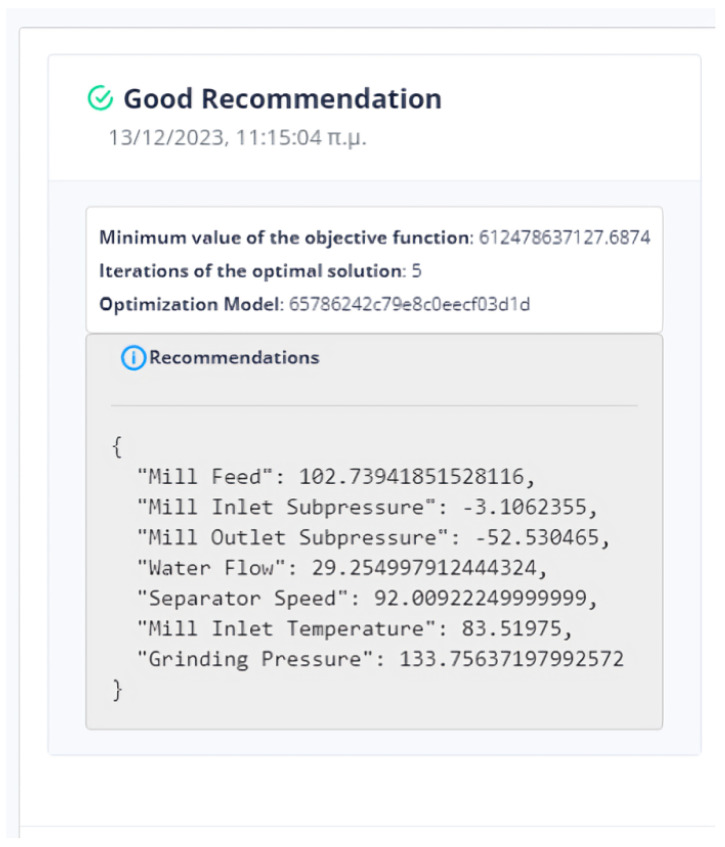
Recommendations of manipulated variables in the first optimization strategy to reach optimal operation in the cement mill.

The evaluation of the optimization is carried out by comparing two variables. The first quantity is the value of the objective function, as obtained by applying the DE method, and the second is the calculation of the objective function by giving the values of the parameters equal to the values at the last data point. If the DE achieves a lower value compared to the last data point, then we have an acceptable recommendation of optimal operation, as in Figure 20.

**Figure 21:**
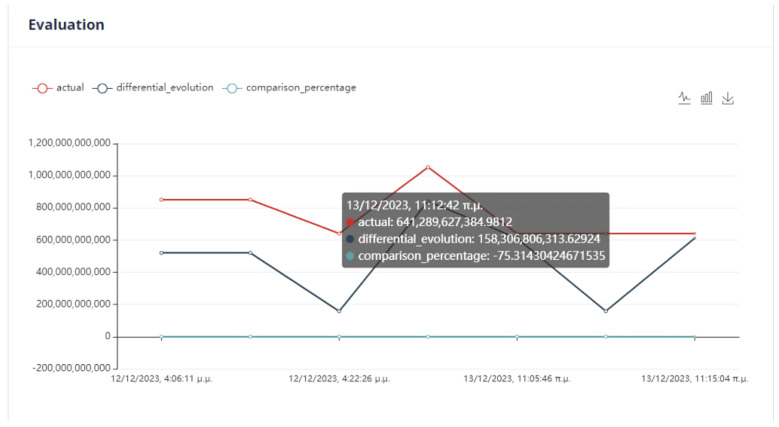
Distribution of recommendations of manipulated variables in the first optimization strategy to reach optimal operation in the cement mill.

Six (6) trials of the DE function were conducted at different times. In all cases, the value of the function from DE is less than the value of the function from the active data point. The actual result of the optimization is the percentage change in the active value of the function relative to the value from DE. In our example, the value of the objective function improves the process by approximately 75%. as is drawn in Figure 21.

**Figure 22:**
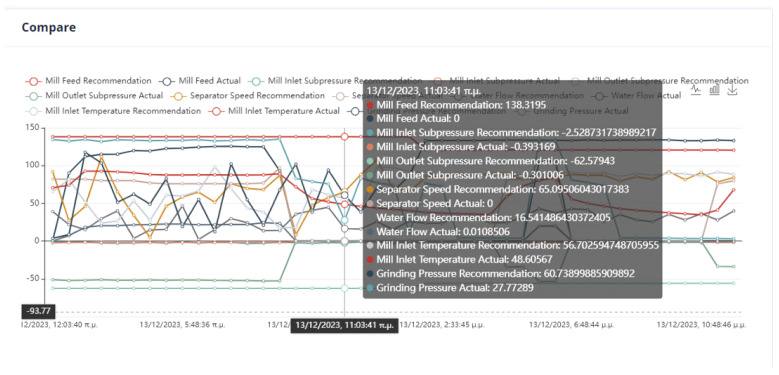
Comparison between actual and recommended values of parameters in the first optimization strategy.

An important issue in the optimization process is the proximity of the proposed values to the effective values so that the system can transition to them without crashing. In Figure 22, it can be seen that the feed of the mill is far from the recommended value. Although in the rest of the cases, the mill will operate without any problems, the possible sudden changes are an issue that must be taken care of by the user.

**Figure 23:**
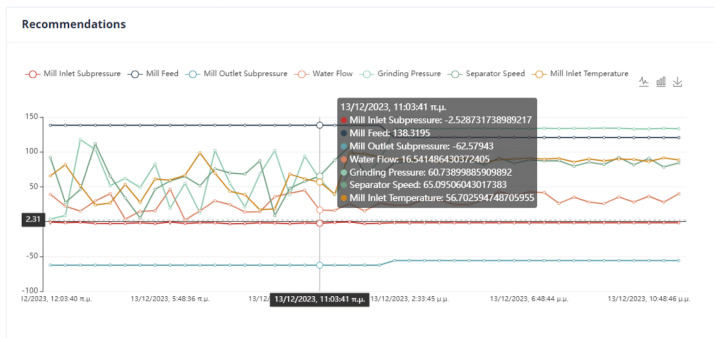
Comparison between actual and recommended values of parameters in the mill in the first optimization strategy.

The system can retain past recommendations and demonstrate them to the user, as in Figure 23. In this way, it is possible to judge indirectly whether the behavior of the system has changed. In addition, the user can determine the time range of the data that will be utilized during the process.

- Second Optimization Strategy

**Figure 24:**
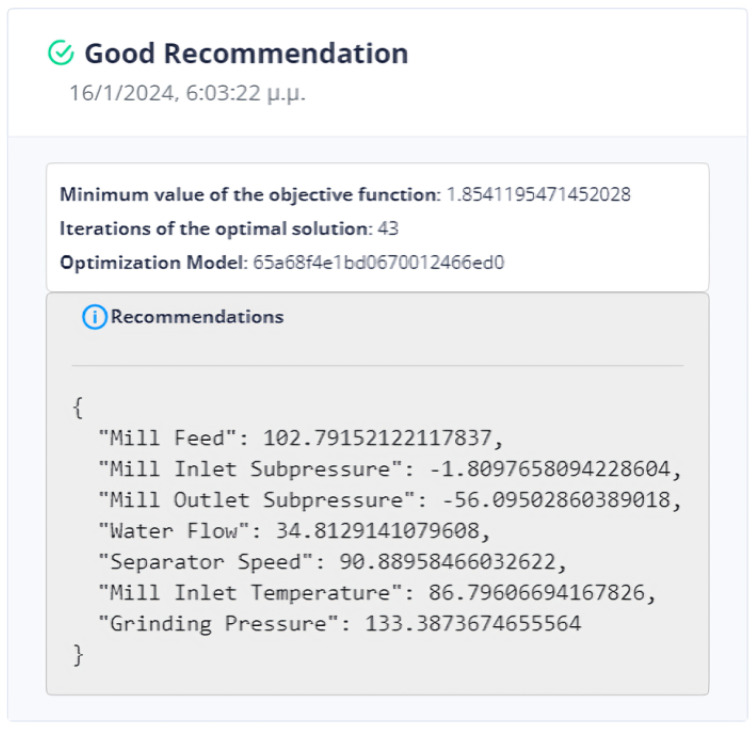
Recommendations of manipulated variables in the second optimization strategy to reach optimal operation in the cement mill.

**Figure 25:**
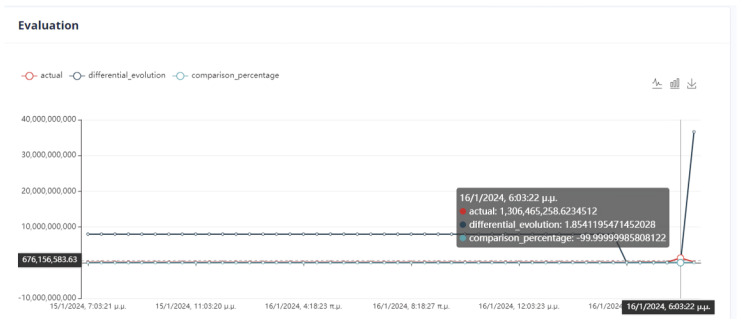
Distribution of recommendations of manipulated variables in the second optimization strategy to reach optimal operation in the cement mill.

**Figure 26:**
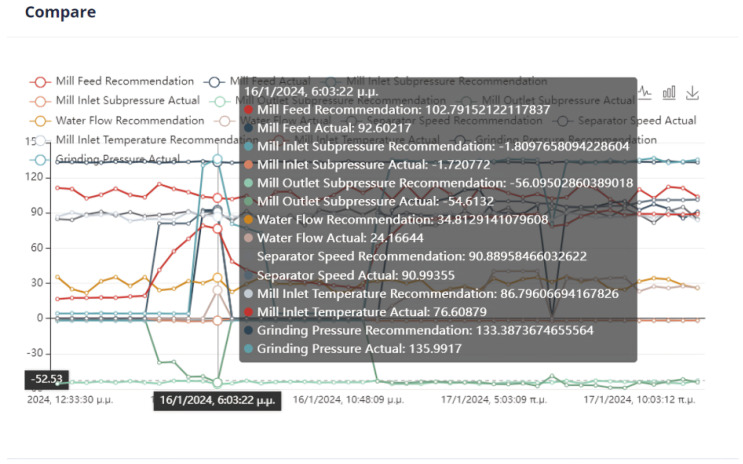
Comparison between actual and recommended values of parameters in the second optimization strategy.

**Figure 27:**
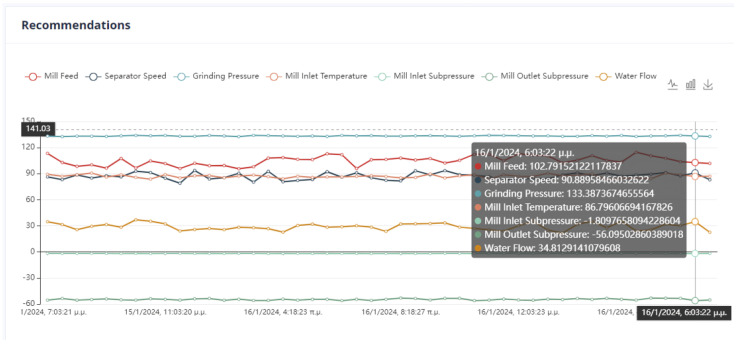
Comparison between actual and recommended values of parameters in the mill in the second optimization strategy.

As we can observe from Figure 24, Figure 25, Figure 26 and Figure 27, it is ensured that the mill can switch to the optimized state as the proposed values are at most thirty percent (30%) higher or lower than the current values. The user can observe the distribution of recommendations and comparisons between the current and recommended values. In our case, the transition to the optimized mode is possible. In essence, the optimization process finds a local optimum where the transition of the cement mill to this cement mode can be made almost immediately.

- Third Optimization Strategy

**Figure 28:**
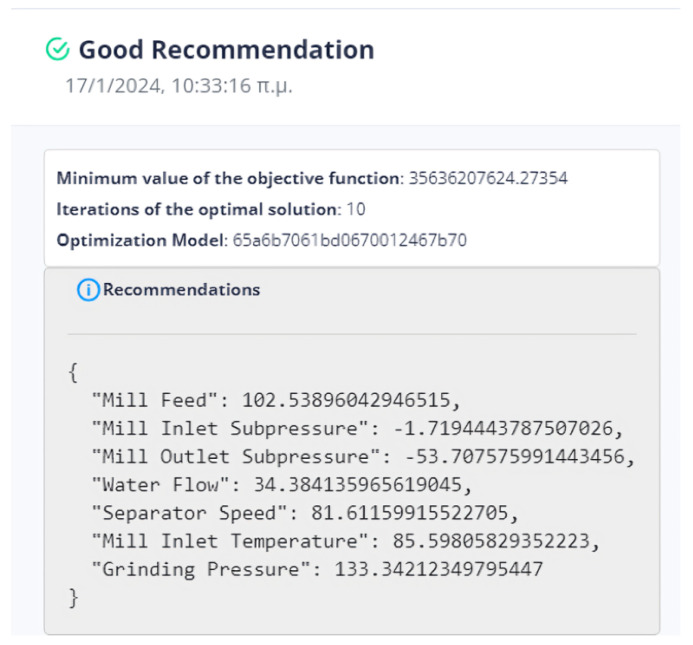
Recommendations of manipulated variables in third optimization strategy to reach optimal operation in the cement mill.

**Figure 29:**
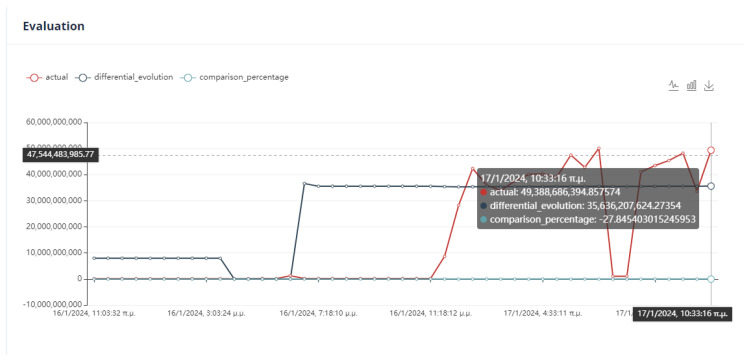
Distribution of recommendations of manipulated variables in third optimization strategy to reach optimal operation in the cement mill.

**Figure 30:**
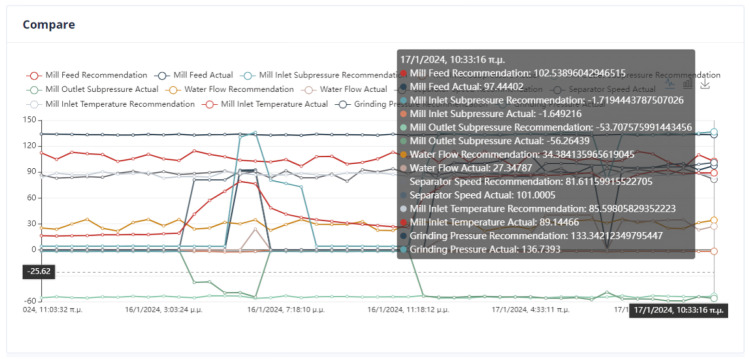
Comparison between actual and recommended values of parameters in third optimization strategy.

**Figure 31:**
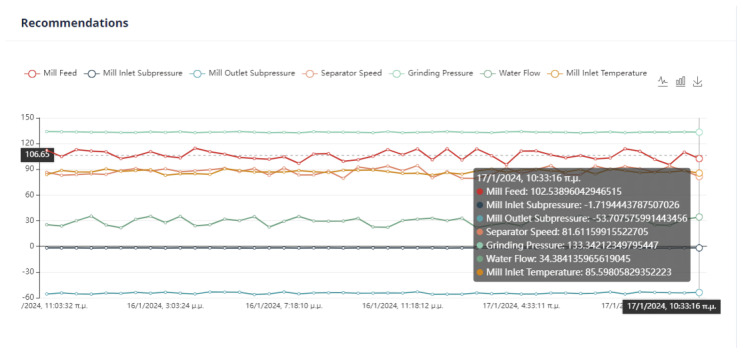
Comparison between actual and recommended values of parameters in the mill in third optimization strategy.

Based on the data presented in Figure 28, Figure 29, Figure 30 and Figure 31, it appears that the transition to the optimized mode was smooth. There was no sudden or abrupt change in the current value and the proposed value of the manipulated variables. In this context, “abrupt change” refers to a percentage difference of more than thirty percent between the current and proposed value. Additionally, the smallest improvement in the current value of the subjective function was observed in this experiment, which was an improvement of only twenty-seven percent. This was the smallest improvement compared to all the other experiments conducted in the mill.

- Fourth Optimization Strategy

**Figure 32:**
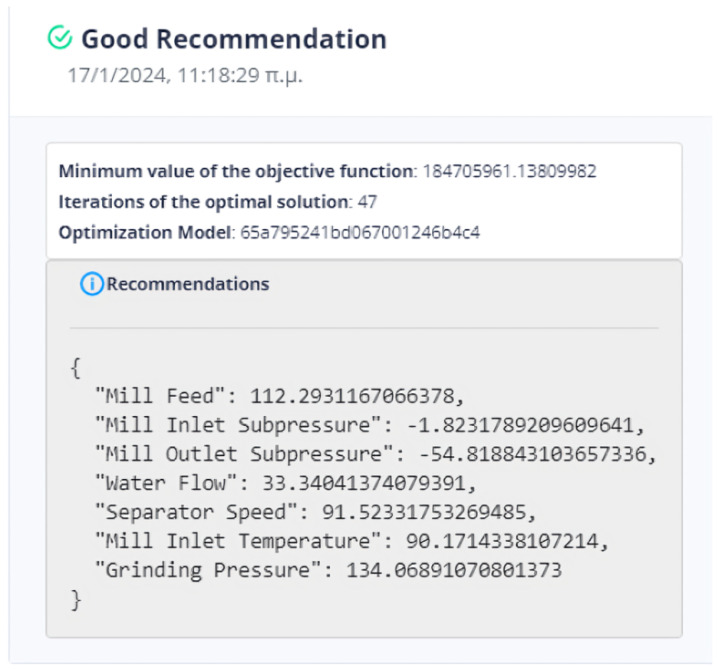
Recommendations of manipulated variables in fourth optimization strategy to reach optimal operation in the cement mill.

**Figure 33:**
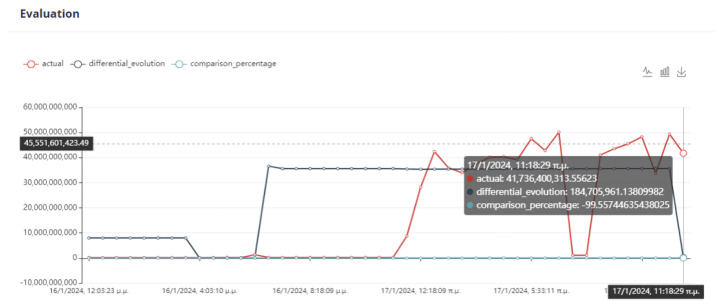
Recommendations of manipulated variables in fourth optimization strategy to reach optimal operation in the cement mill.

**Figure 34:**
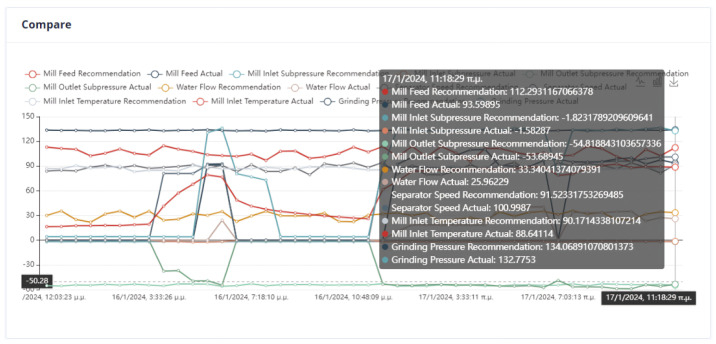
Comparison between actual and recommended values of parameters in fourth optimization strategy.

**Figure 35:**
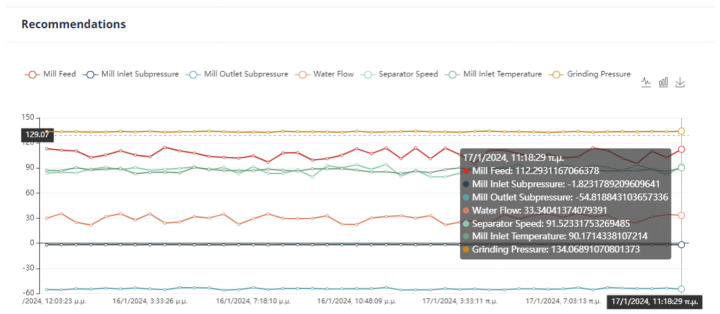
Comparison between actual and recommended values of parameters in the mill in fourth optimization strategy.

In Figure 32, Figure 33, Figure 34 and Figure 35, it is observed that the system suggests feasible values for the covariates. In this case, the production cost is reduced.

#### 4.3.4. The Fourth Optimization Strategy with a User-Defined Model

**Figure 36:**
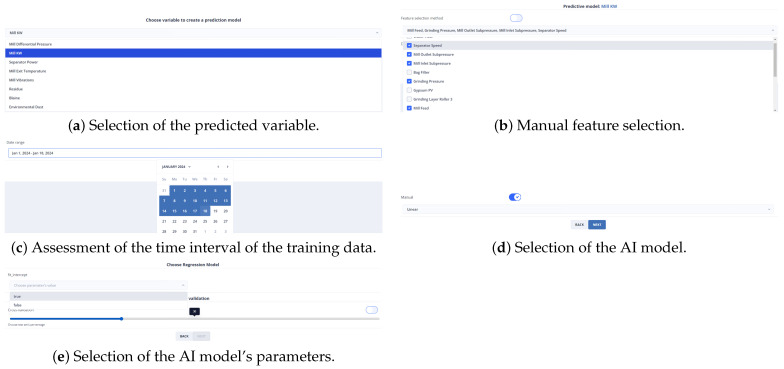
The process of creating an AI model by the user.

Following the fourth optimization strategy, a ML model was developed by configuring the training parameters according to the user’s recommendations instead of relying on a parameter optimization library such as Python’s GridSearchCV. After selecting the non-manipulated variable to be predicted, the features are chosen that are important to predict that variable (Figure 36a,b).

The selection of the mill motor’s energy consumption prediction was based on its high weight of $10^{6}$ in all experiments. Subsequently, the time frame for the training data and the training algorithm is determined, specifically linear regression, as depicted in Figure 36c,d.

The ratio between the training and reduction data is determined based on Figure 36e once the method’s parameters have been selected. Following its training in the backend, this model takes on the active role of predicting the mill’s energy consumption.

**Figure 37:**
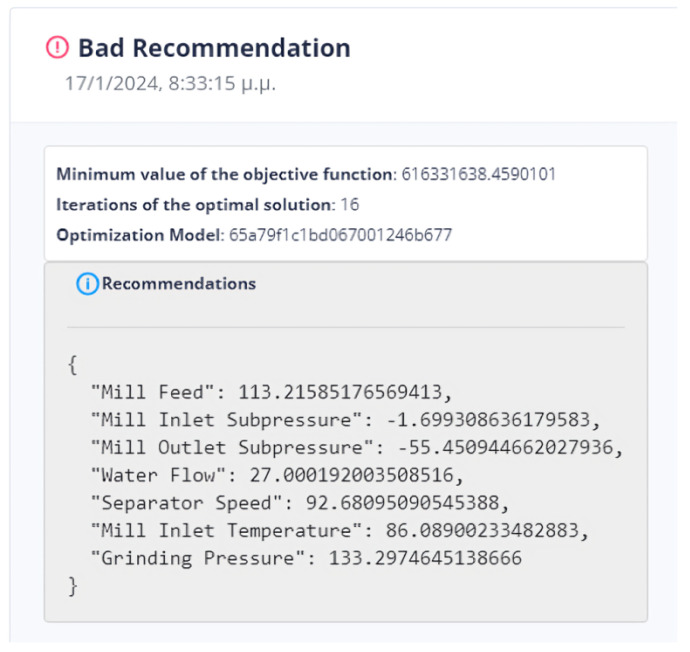
Comparison of the active value of the objective function and the function determined by the DE when the active AI model was created by the user.

**Figure 38:**
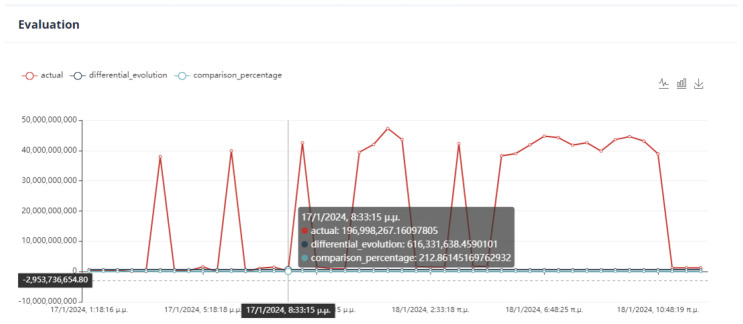
Comparison between actual and recommended values of parameters in the mill in fourth optimization strategy.

The observation made in Figure 37 and Figure 38 indicates that the objective function of DE does not improve but rather worsens in relation to the active value of the objective function. This fact is attributed to its classification as an active prediction model in energy consumption, unlike a random model that has not been verified for optimal performance (i.e., the lowest *NRMSE*). In certain cases, users can determine the active prediction model by connecting specific independent variables to the dependent variable.

### 4.4. Cement Kiln Optimization Results

#### 4.4.1. Experimental Set Up and Optimization Strategies

The kiln is optimized based on both three manipulated variables and three non-manipulated variables predicted by the models. The objective function considers the two types of variables, along with their respective weights. Any fluctuation in the variables that are part of the objective function will be within thirty percent (30%) of its last data value. Specifically, the maximum and minimum values will be thirty percent (30%) greater and less than the last data value, respectively.

There are three optimization strategies for this project. The first strategy considers all variables, both manipulated and non-manipulated. However, it does not prioritize any specific objective, such as reducing energy consumption or environmental impact. The weights of each variable in this strategy are shown in Table 1. The second strategy takes into account the cost of the product and the KPI, which includes kiln amps, total feed, and solid fuel. In this strategy, aside from total feed, all other variables are reduced. The third strategy focuses on environmental impact. The manipulated variables calculated N$O_{x}$, kiln amps, Preheater CO, and solid fuel must all be reduced. The weights of each variable in each strategy are also shown in Table 9.

#### 4.4.2. Setting Parameters from the UI

- First Optimization Strategy

The total feed is given a weight equal to 10 and must be within the limits of the interval [174.285, 323.673]. It is also the only variable that is maximized. The variable Preheater$O_{2}$ shall be within the interval [0.606, 1.126]. It is minimized and obtains a weight of 10. Finally, the solid fuel variable is restricted to the interval [3.963, 7.359], has a weight equal to 10, and is minimized.

**Figure 39:**
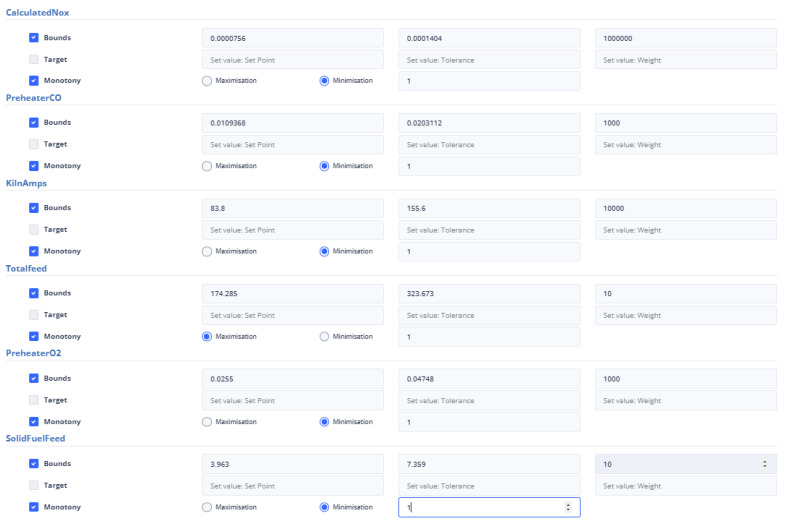
Creation of Objective Function of the 1st Optimization Strategy via the Dashboard.

The calculated NOx variable is minimized and given a weight of 1000. It falls within the range of 0.0000756 and 0.0001404. The kiln amps variable, on the other hand, is also minimized and has a weight of 10,000. It is constrained to be in the range of 83.8 and 155.6. Finally, Preheater CO is minimized with a weight of 1000. It is within the range of 0.0255 and 0.04748. These variables are not manipulated and are bounded within their respective ranges. Figure 39 represents the user interface in that case.

- Second Optimization Strategy

In the optimization process, the last data point used is shown in Figure 6. At this point, the total feed is 229.9054, the solid fuel feed is 5.9962, and the Preheater $O_{2}$ feed is 0.9258. The kiln amps are 119.7, the Preheater CO is 0.05208, and the Calculated NOx is 0.00010855. As mentioned earlier, the optimum value is approximately thirty percent (30%) of the current data value. Based on this assumption, the total feed will be within the range of [160.93, 298.87], the solid fuel feed within [4.1972, 7.7948], Preheater $O_{2}$ within [0.6488, 1.2028], calculated NOx within [756 × $10^{-4}$, 1404 × $10^{-3}$], Preheater CO within [0.0364, 0.0676], and kiln amps within [83.8, 155.6].

**Figure 40:**
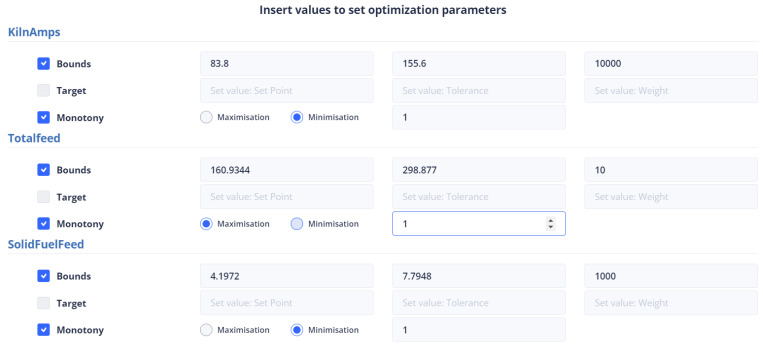
Creation of Objective Function of the 2nd Optimization Strategy via the Dashboard.

The second optimization strategy aims to reduce the production cost of the final product. It involves selecting the conditions that will be included in the final KPI and determined by the UI. This strategy includes kiln amps, total feed, and solid fuel feed, with the weights shown in Table 9. However, the user can set the weights and monotony according to their discretion. The user interface where the constraints and weights of the objective function are given is shown in Figure 40.

- Third Optimization Strategy

**Figure 41:**
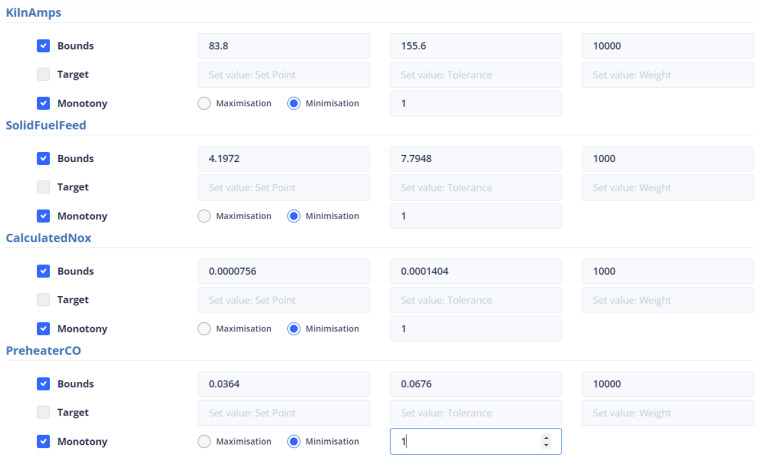
Creation of Objective Function of the 3rd Optimization Strategy via the Dashboard.

The third strategy for optimization considers the data point from the second strategy. This strategy pertains to enhancing the environmental impact of the kiln. In this respect, it is essential that the dynamic objective function includes variables with environmental implications. These are calculated NOx, Preheater CO, solid fuel feed and kiln amps. The weights given to the variables are shown in Table 9. Energy consumption, particularly kiln amps, plays a crucial role in this strategy, as well as in previous and future strategies. The configuration of the parameters from the user interface is shown in Figure 41.

In the kiln, the operator makes the corresponding settings to the dashboard in the mill shown in Figure 13, Figure 14 and Figure 19.

#### 4.4.3. Cement Kiln Results

- First Optimization Strategy

**Figure 42:**
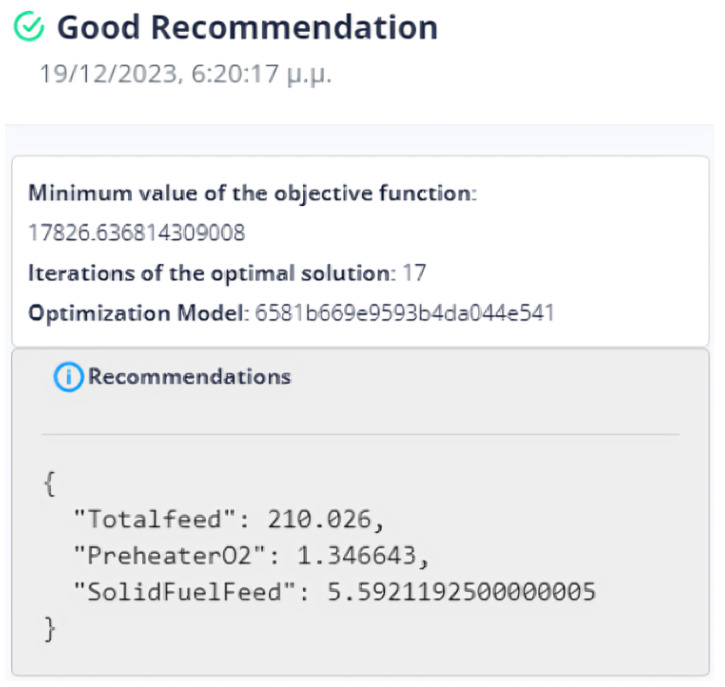
Proposed optimal values for the manipulated variables to attain optimal operation in the cement kiln in the first optimization strategy.

**Figure 43:**
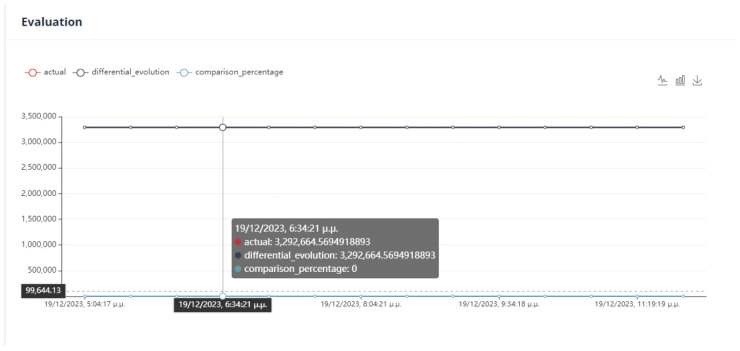
The quantitative difference between the active value of the objective function and the value explained by the DE in the first optimization strategy.

Proposed optimal values in the first optimization strategy are shown in Figure 42. The objective function does not improve in relation to the last value from the last data point, as observed in Figure 43. What the objective function achieves, however, is to set the variables within the predefined intervals or constraints.

**Figure 44:**
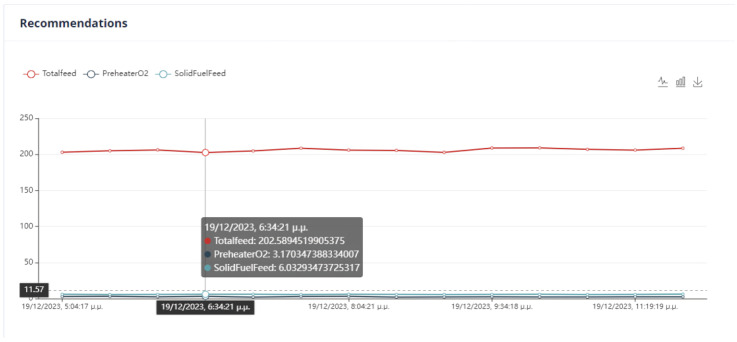
The distribution of system’s recommendations in kiln in the first optimization strategy.

The system’s recommendations to the user are recorded in the dashboard as shown in Figure 44. The application user can refer to earlier system recommendations where another objective function was probably involved in the optimization.

**Figure 45:**
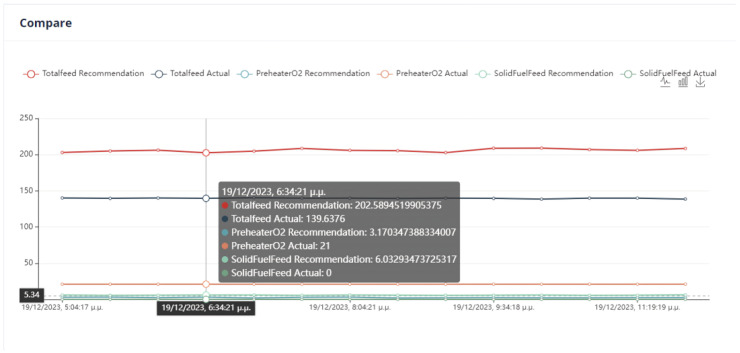
Comparison between actual and recommended values of parameters in the kiln in the first optimization strategy.

Observing Figure 45, it can be concluded that the recommendations optimize the system. For example, the total feed is increased relative to the current value. The user has to decide if it is feasible to switch to this production point. The same is observed for the Preheater $O_{2}$ variable, where the active value is higher than the recommended value.

- Second Optimization Strategy

**Figure 46:**
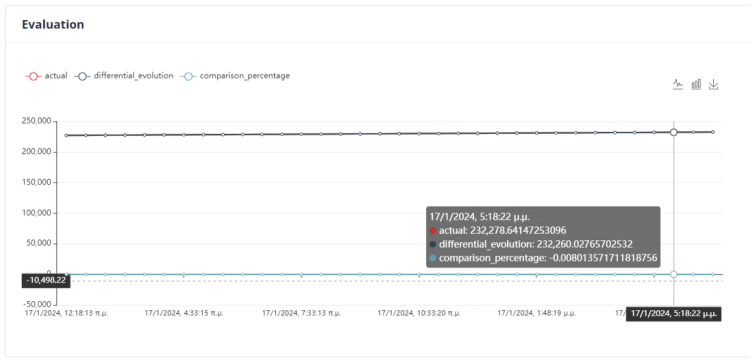
The quantitative difference between the active value of the objective function and the value explained by the DE in the second optimization strategy.

In this case, we have a very small improvement in the objective function from DE compared to the current value from the last data point, as illustrated in Figure 46. It can be seen that the models need to be retrained with new data. A limitation of this work is the lack of a retraining system. The programmer, in case he sees different behavior, resorts to the k-means algorithm in order to resort to the background of the application to retrain the AI models.

**Figure 47:**
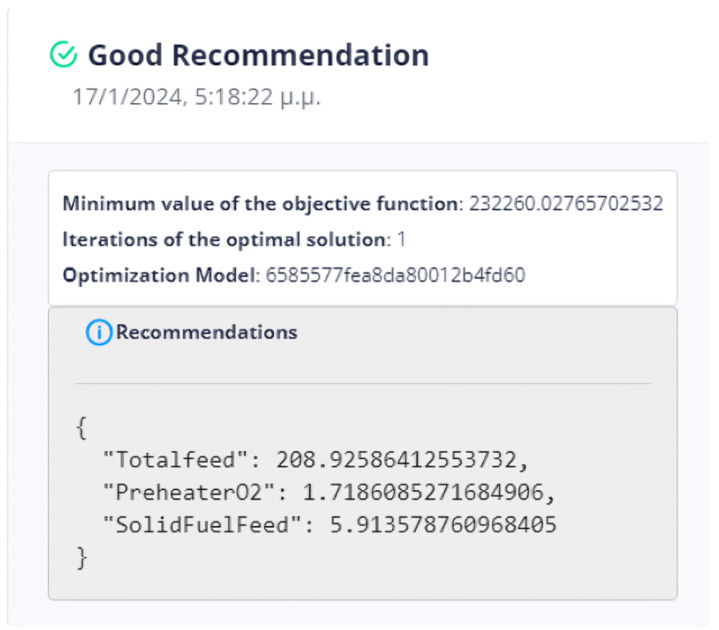
Proposed optimal values for the manipulated variables to attain optimal operation in the cement kiln in the second optimization strategy.

**Figure 48:**
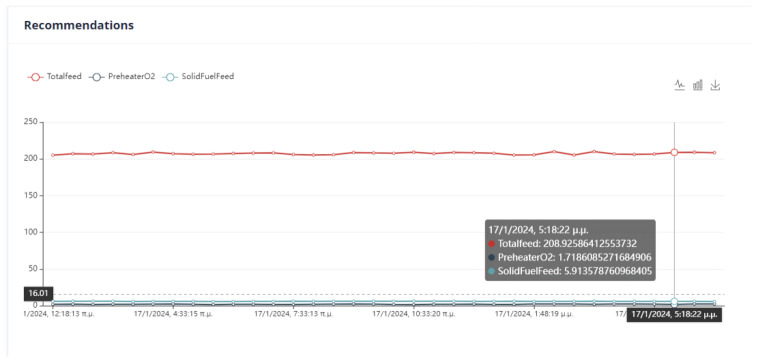
The distribution of system recommendations in the kiln in the second optimization strategy.

**Figure 49:**
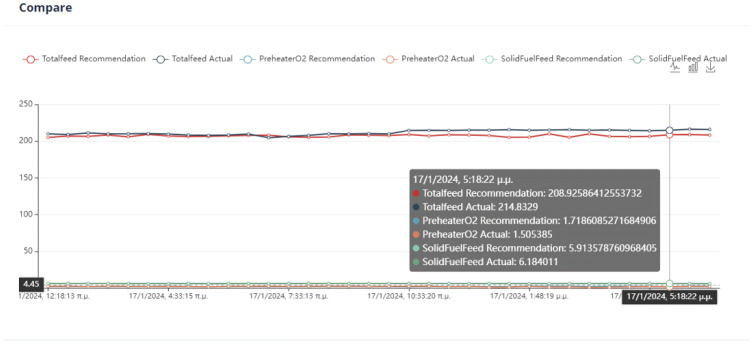
Comparison between actual and recommended values of parameters in the kiln in the second optimization strategy.

Observing Figure 40, Figure 47, Figure 48 and Figure 49, the variables total feed and solid fuel feed are within the constraints set by the user. These recommendations are acceptable since, in addition to optimizing the KPI, they ensure that the transition of the kiln to this mode of operation is possible.

- Third Optimization Strategy

**Figure 50:**
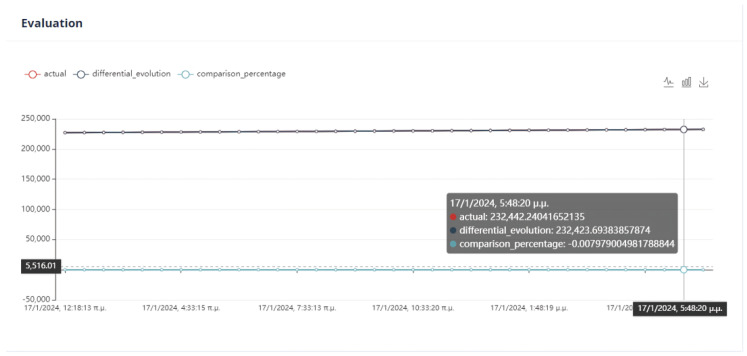
The quantitative difference between the active value of the objective function and the value explained by the DE in the third optimization strategy.

As in the second optimization strategy, in the third strategy concerning the environmental footprint, the improvement from the DE, as can be observed in Figure 50, relative to the current value of the objective function resulting from replacing the values of the last data point, is infinitesimal. The greatest likelihood of this phenomenon occurring is the need to retrain the AI models.

**Figure 51:**
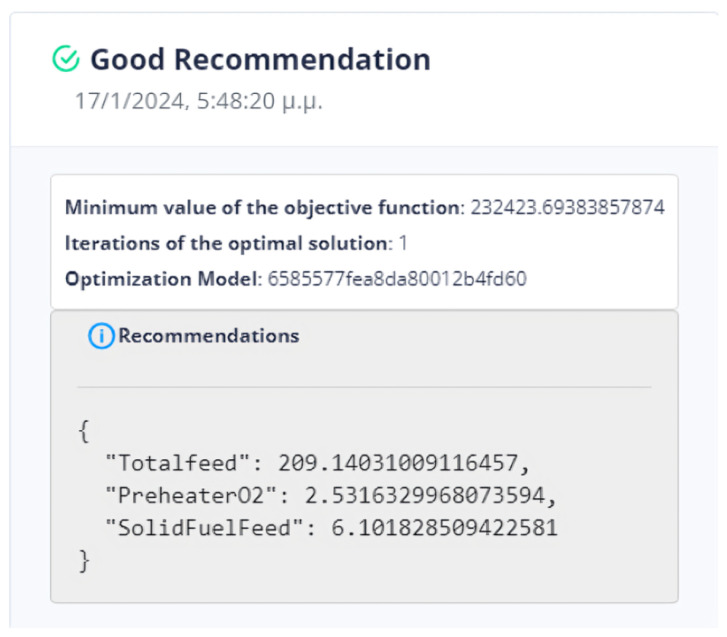
Proposed optimal values for the manipulated variables to attain optimal operation in the cement kiln in the third optimization strategy.

**Figure 52:**
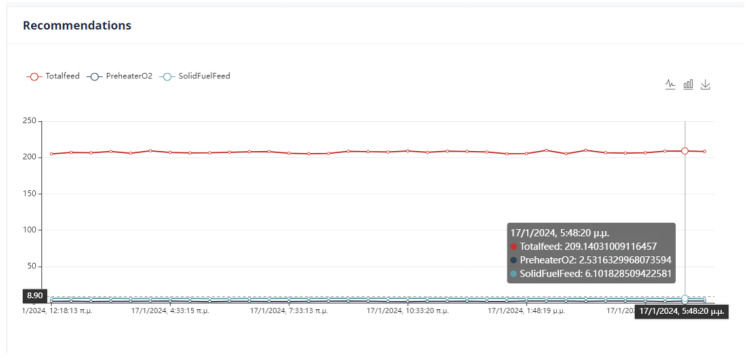
The distribution of system recommendations in the kiln in the third optimization strategy.

**Figure 53:**
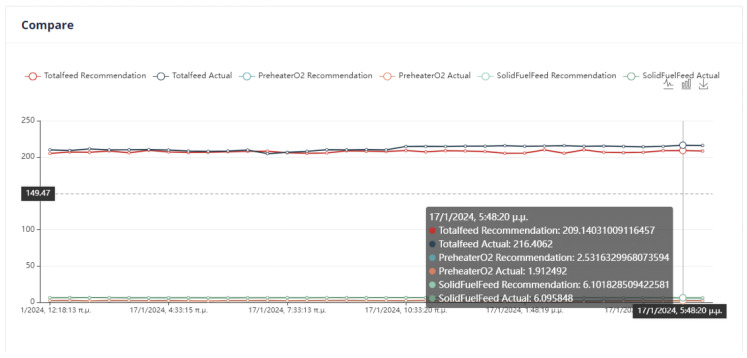
Comparison between actual and recommended values of parameters in the kiln in the third optimization strategy.

By examining Figure 41, Figure 51, Figure 52 and Figure 53, it can be determined that the variable solid fuel feed adheres to the user-defined constraints. These recommendations are acceptable since, in addition to optimizing the KPI, the operator ensures that the transition of the kiln to this mode of operation is possible.

## 5. Discussion

The cement industry is presently one of the most energy-intensive sectors. In addition, cement is an important material in the construction sector. It is essential to combine with the maintenance of the quality of cement expressed by the Blaine and residue indices, the reduction of energy consumption, and the devaluation of the environmental footprint of the plants. The cement production has many parameters that are either calculated by deterministic functions or measured by special sensors. Furthermore, during production, there are either manipulated variables that can be defined by the cement plant user, non-manipulated variables, or uncontrolled variables. The non-manipulated variables play an important role in the optimization of the production, and the uncontrollable variables are obtaining values within a predefined range. The non-manipulated variables of either the mill or the kiln depend linearly or non-linearly on both the manipulated variables and the uncontrollable variables. The relationships between these parameters are difficult to determine, so feature selection methods are used to find the most relevant parameters. ML models are then trained to incorporate the correlation between each dependent variable and independent variables.

The current study concerns a cement industry in Greece that has several plants. Each cement plant has different stresses, and the maintenance works of each machine change the data distribution of each parameter. For the above reasons, it is imperative to retrain the AI models if the production conditions change. However, many times, the data distribution changes as the production conditions are dynamic, e.g., there are parameters such as internal or external temperature. In this work, the expert is concerned with the data distribution, and if a sharp concept shift is observed, the data are clustered into groups and retrained. The overall *NRMSE* resulting from all clusters is the final measure. With the data provided by the factory, no improvement in the results of the cement mill models was observed. In addition, no improvement was observed in any of the predicted variables of the kiln. The optimization of the production parameters in the cement mill and the cement kiln was derived from a dynamic objective function in which the expert operator can assign monotony or value-weighting to each manipulated or non-manipulated variable. According to the optimization strategies that came from the operators of the cement plant, the objective function is improved compared with the value that obtains the objective function if the values of the variables came from the last database entry.

A major limitation of the work is the lack of many different advanced clustering techniques other than k-means to which we could compare their performance. This limitation could be extended to the case of the AI subsystem, where the introduction of more ML or DL methods would ensure the robustness of the prediction models. Another major limitation is the absence of an autonomous agent that calculates the concept drift of data. If the calculations exceed a threshold, it would trigger the retraining process. The triggering of the retraining of the AI models currently relies upon user’s experience by assessing the distribution drift of the data. However, this approach can potentially lead to delays in the activation of retraining and may result in errors on the part of the user. It is important to note that there can be a limitation when it comes to converting the current operating values of a mill or kiln to the proposed values obtained from the IDMS. This conversion cannot be done abruptly, and it is the responsibility of the system user to handle this issue.

## 6. Conclusions

In cement production, there are two primary units in the plant: the mill and the kiln. The plant variables are divided into manipulated and non-manipulated. In this work, the optimization method was combined with feature selection algorithms, AI methods, and behavioral data separation. The AI models provide non-manipulated variables that are dependent on either manipulated or non-controlled variables. In the context of a cement plant in Greece, the AI models were trained to identify the best model for each non-manipulated variable based on the *NRMSE* metric. During the initial training, only a few variables showed *NRMSE* below 0.5 for their models. These variables were energy consumption, differential pressure, exit temperature in the mill, and amperes in the kiln. The kiln feed data demonstrated two distinct behaviors, which allowed for the creation of weighted models. However, it was observed that the NRNSE either did not improve or improved slightly. The best-performing model with the best set of parameters was chosen among MLP, GB, LGBM, XGBoost, KNN, linear regression, TTR and CatGBoostRegressor.

The non-linear relationships between the manipulated and non-manipulated variables were represented by incorporating the models into the DE optimization function. The important features of the database for each trained model were derived from feature selection algorithms, more specifically, RFECV and SFS. Often, the selection of important features for an AI model does not align with the knowledge that cement engineers have about the relationships between dependent and independent variables. To address this, the proposed system allows users to manually define the AI model. The objective function had a linear form and contained all the non-manipulated variables of the cement plant. Various factors can impact the environmental footprint of the manufacturing process, e.g., environmental dust in the mill and Calculated NOx in the kiln. In addition, energy consumption can be influenced, e.g., by mill motor energy consumption and kiln amps, while production costs may be affected, e.g., by external temperature in the mill and kiln fuel supply. Moreover, the quality of the product can be influenced by the Blaine and residue indices present in the mill.

A major innovation was the definition of the objective function by the user, where each selection from the dashboard created an additional term in a linear form function. The non-linear relationships of the functions were expressed by the AI models. More specifically, a dynamic system was created to fulfill the factory management’s desire for optimizing different parameters. This system allows the user to determine the weight, constraints, target values, or monotony of each variable depending on their requirements. To avoid abrupt transitions in operation that would lead to the malfunction of the machines, the constraints of the non-manipulated variables were specified to be within ten percent (10%) to thirty percent (30%) from the active variable. The system disables variables that are not of interest to the user, so they receive zero weight when not selected. This creates a dynamic objective function that can be adapted to different optimization strategies, for example, reducing energy consumption. Depending on the optimization strategy used, the value of the objective function is converted into a KPI every time. The system evaluates the optimized value of the objective function by conducting experiments according to the designated strategies and only considers it permissible if it meets the required criteria. In most cases, the final results depicted in the dashboard showed that the value of the objective function decreases with the application of DE concerning the active value of the objective function from the last data point in both the mill and the kiln of the cement plant.

However, the manual model for energy consumption in the mill and the kiln models, which have an *NRMSE* close to one (1), have led to unacceptable optimization and marginally acceptable optimization, respectively. This sentence implies that the main focus of future work will be on data behavior and how to handle it. Instead of relying on the user’s intuition, which can lead to delays and poor estimation, an autonomous agent will be embedded in the system to signal when retraining of the models is required due to data drift. Furthermore, in a future extension of the work, more ML or deep learning (DL) models will be incorporated in order to allow the choice between different combinations of models and parameters to lead to models with *NRMSE* close to zero (0).

## Figures and Tables

**Table 1:** Overview of the relevant works. The checkmark point indicates that the method is relevant to the category indicated in the corresponding column.

Reference	Cement Mill Optimization	Cement Kiln Optimization	Optimization of Other Functionality	Mathematical Optimization Method	Improvement of Architecture and/or Control Process	Method
[7]	-	-	✓	✓	-	GA
[18]	-	✓	-	✓	-	ANFIS-GA
[20]	-	✓	✓	-	✓	HRSO
[21]	-	✓	✓	-	✓	PM-NOx concentration
[22]	-	✓	✓	-	✓	CFD
[23]	✓	✓	✓	✓	-	GA
[24]	-	✓	✓	-	✓	RMCM
[25]	-	-	✓	✓	✓	Introducing nano-Si$O_{2}$
[26]	-	-	✓	✓	✓	Creation of DA-PEG
[27]	-	✓	-	✓	-	PC - SCN - SSA
[28]	✓	-	✓	✓	-	MOBAS
[29]	✓	-	✓	✓	-	BBD
[30]	✓	✓	✓	✓	-	BPNN - MOBAS
[31]	✓	-	✓	✓	-	PSOA
[32]	✓	-	✓	✓	-	BO-LightGBM
[33]	✓	✓	✓	✓	-	BDA
[34]	✓	✓	✓	✓	-	BDA
[35]	✓	✓	✓	-	✓	PLC
[36]	✓	-	-	-	✓	APC
[37]	✓	✓	✓	-	✓	IS
[38]	-	✓	-	✓	-	SAAS, AI, APC
[39]	✓	-	-	✓	-	AI, DE
Our Approach	✓	✓	-	✓	-	AI, DE

**Table 2:** The selected features in cement mill for each variable.

Dependent Variable Y	Independent Variables	Manipulated Variables
Mill Motor	Grinding Pressure, Separator Speed, Mill Feed, Water Flow, Mill Inlet Pressure, Limestone%, Pozzolana%, FlyAsh%, Grinding Aid PV, Grinding Layer Roller	Grinding Pressure, Separator Speed, Mill Feed, Water Flow, Mill Inlet Pressure
Mill Differential Pressure	Mill Feed, Mill Outlet Pressure, Bag Filter, Mill Inlet Pressure, Separator Speed, Gypsum%	Mill Feed, Mill Outlet Pressure, Mill Inlet Pressure, Separator Speed
Separator Motor	Mill Feed, Separator Speed, Mill Outlet Pressure, Grinding Pressure, Mill Inlet Temperature, Limestone%, Pozzolana%, Fly Ash%, Grinding Layer Roller	
Mill Exit Temperature	Mill Feed, Grinding Pressure, Separator Speed, Mill Inlet Temperature, Mill Inlet Pressure, Limestone%, Pozzolana%, FlyAsh%	Mill Feed, Grinding Pressure, Separator Speed, Mill Inlet Temperature, Mill Inlet Pressure
Environmental Dust	Separator Speed, Bag Filter, Limestone%	Separator Speed
Blaine	Mill Outlet Pressure, Limestone%, FlyAsh%	Mill Outlet Pressure
Residue	Mill Inlet Pressure, Mill Outlet Pressure, Water Flow, Limestone%	Mill Inlet Pressure, Mill Outlet Pressure, Water Flow
Mill Vibrations	Mill Feed, Mill Inlet Pressure, Water Flow, Separator Speed, Grinding Pressure, Limestone%	Mill Feed, Mill Inlet Pressure, Water Flow, Separator Speed, Grinding Pressure

**Table 3:** The selected features in Kiln for each variable.

Dependent Variable Y	Independent Variables	Manipulated Variables
Calculated NOx	Heat Main Burner, Clinker $C_{a}$O, Kiln Feed LSF, Total Feed, Preheater$O_{2}$, Solid Fuel Feed	Preheater$O_{2}$, Solid Fuel Feed, Total Feed
Kiln Amps	Total Air Flow, Secondary Air Temp, Kiln Vortex Temp, Solid Fuel Feed, Kiln Inlet Press, Total Feed, Preheater$O_{2}$	Preheater$O_{2}$, Solid Fuel Feed, Total Feed
Preheater CO	Kiln Inlet Press, Press Transport Air, Press MAS Air, Total Air Flow, Clinker $C_{a}$O, Secondary Air Temp, Total Feed, Preheater$O_{2}$, Solid Fuel Feed	Preheater$O_{2}$, Solid Fuel Feed, Total Feed

**Table 4:** The results of supervised learning models for the cement mill.

Variable	Model Type	Number of Estimators	Max Depth	Number of Leaves	*NRMSE*
Mill Motor	RF	10	5	-	0.34
Mill Differential Pressure	LGBM	-	10	10	0.23
Separator Motor	LGBM	-	5	12	0.49
Mill Exit Temperature	XGBoost	50	3	-	0.35
Environmental Dust	GBR	50	3	-	0.89
Blaine	GBR	50	3	-	0.67
Residue	TTR	-	-	-	0.78
Mill Vibrations	RF	50	5	-	0.69

**Table 5:** The results of supervised learning models for the kiln.

Variable	Model Type	Fit Intercept	Number of Estimators	Max Depth	Hidden Layer Sizes	Number of Leaves	*NRMSE*
Calculated NOx	MLP	-	-	-	(5,)	-	0.95
Kiln Amps	XGBoost	-	50	3	-	-	0.14
Preheater CO	LGBM	-	-	5	-	8	0.96

**Table 6:** Results of the cement mill’s supervised learning models when the data are clustered.

Variable	Model Type	Number of Estimators	Max Depth	Number of Leaves	Hidden Layer Sizes	*NRMSE*
Mill Motor	RF	50	10	-	-	0.40
Mill Differential Pressure	MLP	-	-	-	(5,)	0.21
Separator Motor	XGBoost	50	3	-	-	0.53
Mill Exit Temperature	XGBoost	50	3	-	-	0.37
Environmental Dust	LGBM	-	5	8	-	0.84
Blaine	MLP	-	-	-	(5,)	0.68
Residue	TTR	-	-	-	-	0.76
Mill Vibrations	LGBM	-	5	10	-	0.70

**Table 7:** Results of the cement kiln’s supervised learning models when the data are clustered.

Variable	Model Type	Number of Estimators	Max Depth	Number of Leaves	*NRMSE*
Calculated NOx	TTR	-	-	-	0.99
Kiln Amps	XGBoost	50	3	-	0.21
Preheater CO	GBR	50	3	-	0.98

**Table 8:** Optimization strategies and weights of each factor in the cement mill.

Optimization Strategy	Main Target	Mill Feed Weight	Mill KW Weight	Separator Power Weight	Vibrations Weight	Mill DP Weight	Environ. Dust Weight	Mill Exit Temperature Weight	Blaine Weight	Residue Weight
1st	Combined Optimal	1 (increase)	$10^{6}$ (decrease)	$10^{5}$ (decrease)	$10^{6}$ (decrease)	$10^{4}$ (increase)	$10^{2}$ (decrease)	$10^{3}$ (increase)	10 (Target)	10 (Target)
2nd	Energy Optimal	$10^{3}$ (increase)	$10^{6}$ (decrease)	$10^{5}$ (decrease)	-	-	-	-	-	-
3rd	Environmental Impact	-	$10^{6}$ (decrease)	$10^{5}$ (decrease)	-	-	$10^{5}$ (decrease)	$10^{3}$ (increase)	-	-
4th	Production Cost	$10^{2}$ (increase)	$10^{6}$ (decrease)	$10^{5}$ (decrease)	-	-	-	-	$10^{3}$ (Target)	$10^{2}$ (Target)

**Table 9:** Optimization Strategies and weight of each factor in cement kiln.

Optimization Strategy	Main Target	Calculated NOx	Kiln Amps	Preheater CO	Total Feed	Preheater $O_{2}$	Solid Fuel Feed
1st	Combined Optimal	$10^{6}$ (decrease)	$10^{4}$ (decrease)	$10^{3}$ (decrease)	10 (increase)	$10^{3}$ (decrease)	10 (decrease)
2nd	Production Cost	-	$10^{4}$ (decrease)	-	10 (increase)	-	$10^{3}$ (decrease)
3rd	Environmental Impact	$10^{3}$ (decrease)	$10^{4}$ (decrease)	$10^{4}$ (decrease)	-	-	$10^{3}$ (decrease)

## Data Availability

All data that is available for public sharing is contained within the article.

## Abbreviations

The following abbreviations are used in this manuscript:

AI	Artificial intelligence
AHP	Analytic hierarchy process
ANFIS	Adaptive neuro-fuzzy inference systems
APC	Advanced process control
BBD	Box–Behnken design
BDA	Big data analytics
BO-LightGBM	Bayesian optimization light gradient boosting machine
BPNN	Back propagation neural network
CCS	Cubic compressive strength
CFD	Computational fluid dynamics
C-TAEA	Constrained–targeted adaptive evolutionary algorithm
DA-PEG	Decanoic acid/polyethylene glycol binary eutectic
DE	Differential evolution
DL	Deep learning
DP	Differential pressure
DT	Decision tree
ERP	Enterprise resource planning

FIS	Fuzzy inference system
GA	Genetic algorithm
GB	Gradient boosting regressor
GP	Goal programming
HL	High limit
HRSO	Heat recovery system optimization
IDMs	Intelligent decision making system
IoT	Internet-of-Things
IS	Information system
KNN	k-nearest neighbors
KPIs	key performance indicators
KW	Kilowatt
LGBM	Light gradient boosting regressor
LL	Low limit
MPCC	Magnesium phosphate cementitious composites
MDPI	Multidisciplinary Digital Publishing Institute
ML	Machine learning
MLP	Multi-layer perceptron regressor
MOBAS	Multi-objective beetle antennae search
MQTT	Message queuing telemetry transport
NOx	Nitric oxides
*NRMSE*	Normalized root mean squared error
PC	Principal components
PCMs	Phase change materials
PLC	Programmable logic controller
PM	Precalciner model
PSD	Particle size distribution
PSO-BP-ANN	Back propagation artificial neural network
PV	Process value
REST API	RESTful application programming interface
RFECV	Recursive feature elimination with cross-validation
PM	Particulate matter
PSOA	Particle swarm optimization algorithm
RMCM	Raw material carbonate method
SAAS	Software as a Service
SCN	Stochastic configuration networks
SFS	Sequential forward selection
Si$O_{2}$	Silicon dioxide
SP	Set point
SSA	Hammersley-based salp swarm algorithm
SVM	Support vector machine
TOPSIS	Technique for order of preference by similarity
TTR	Transformed target regressor
UI	User interface
XGBoost	Extreme gradient boosting regressor
